# Rapid de novo evolution of lysis genes in single-stranded RNA phages

**DOI:** 10.1038/s41467-020-19860-0

**Published:** 2020-11-26

**Authors:** Karthik R. Chamakura, Jennifer S. Tran, Chandler O’Leary, Hannah G. Lisciandro, Sophia F. Antillon, Kameron D. Garza, Elizabeth Tran, Lorna Min, Ry Young

**Affiliations:** 1 Center for Phage Technology and Texas A&M AgriLife, Department of Biochemistry and Biophysics, Texas A&M University, College Station, TX 77843-2128 USA; 2 Present Address: Pharmaceutical Sciences Division, University of Wisconsin-Madison, Madison, WI 53705 USA; 3 Present Address: University of North Texas Health Science Center, Fort Worth, TX 43210 USA; 4 Present Address: College of Pharmacy, University of North Texas Health Science Center, Fort Worth, TX 43210 USA; 5 Present Address: Baylor College of Medicine, Houston, TX 77030 USA

**Keywords:** Evolutionary genetics, Evolutionary biology, Bacteriophages

## Abstract

Leviviruses are bacteriophages with small single-stranded RNA genomes consisting of 3-4 genes, one of which (*sgl*) encodes a protein that induces the host to undergo autolysis and liberate progeny virions. Recent meta-transcriptomic studies have uncovered thousands of leviviral genomes, but most of these lack an annotated *sgl*, mainly due to the small size, lack of sequence similarity, and embedded nature of these genes. Here, we identify *sgl* genes in 244 leviviral genomes and functionally characterize them in *Escherichia coli*. We show that leviviruses readily evolve *sgl* genes and sometimes have more than one per genome. Moreover, these genes share little to no similarity with each other or to previously known *sgl* genes, thus representing a rich source for potential protein antibiotics.

## Abstract (web summary)

Leviviruses are phages with ssRNA genomes that encode a protein (Sgl) that induces host autolysis by interfering with bacterial cell wall synthesis. Identification of *sgl* genes is complicated by their small size and lack of sequence similarity. Here, Chamakura et al. use bioinformatic and experimental approaches to identify *sgl* genes in 244 leviviral genomes.

## Introduction

Each levivirus has three core genes, two of which encode proteins of the *T* = 3 icosahedral shell: Coat (178 copies) and Mat (1 copy)^1–3^ (Supplementary Fig. 1). All known leviviruses use a retractable pilus as a receptor, recognized and bound by Mat^4,5^. The two best-studied leviviruses are MS2 and Qβ, both specific for the F conjugational pilus in *E. coli*. The Sgl of MS2 is L, a 75 aa product of a reading frame overlapping the end of *coat* and the beginning of *rep*^6^ (Supplementary Fig. 1). In Qβ, the Mat protein, called A_2_ for historical reasons, moonlights as the Sgl^7^. The lytic function of A_2_ derives from its direct non-competitive inhibition of MurA, the first enzyme in the peptidoglycan (PG) biosynthesis pathway^8^. The lytic function of L is not understood mechanistically, other than it requires the chaperone DnaJ and does not involve inhibition of PG biosynthesis^9,10^. Until recently, only eight other *Leviviridae* (not counting 25 close relatives of MS2 and Qβ) have been isolated and subjected to genomic analysis (Supplementary Table 1). These phages are specific for a wide range of retractable conjugational and motility pili^11,12^. In seven of the eight cases, the *sgl* was identified by cloning and testing in *E. coli*;^13,14^ no *sgl* has been identified in the eighth^15^. We have recently shown that the Sgl of M, a levivirus specific for the IncM conjugational pilus, blocks PG biosynthesis by inhibiting MurJ, the lipid II flippase^16^ (Supplementary Fig. 1 and Supplementary Table 1). Five of the phages have small Sgls that, although not detectably similar to MS2 L, have an L-like motif architecture that we have recently identified through genetic analysis^17^ (Supplementary Fig. 1). Four of these *sgl*s are located roughly at the same genomic position as L, but one, L^AP205^, is encoded at the extreme 5′ end of the gRNA^14^. Taking the simplest notion, that the six L-like Sgls attack the same target (Supplementary Table 1), finding additional protein antibiotic genes has had a very limited horizon, with the sole remaining Sgl, encoded by the *C. crescentus* phage Cb5, already under investigation in this laboratory. However, in 2016, the tally of total ssRNA genomes deposited in the NCBI database increased by more than 10-fold as a result of a search for ssRNA phage genomes in various metatranscriptomes^18,19^. Unfortunately, only one of these (AVE017) had an annotated lysis gene; in this case, the Sgl shared ~38% sequence similarity to MS2 L (Supplementary Fig. 1). Even more recent studies have uncovered tens of thousands of leviviral genomes, highlighting the incredible diversity of ssRNA phages in the environment^20,21^.

In this work, we conducted a systematic experimental search for Sgls in the leviviral genomes. The results provide insights into not only the diversity of Sgl proteins but also into the evolution of genes.

## Results

### Finding candidate *sgl*s and phenotypic analysis

Without any BLAST hits to known Sgls other than AVE017, we devised a strategy to identify the potential *sgl* candidates in these recently discovered ssRNA phage genomes without reliance on homology searches. The first step was arbitrarily setting the minimum ORF length to 25 codons; this led to >10 candidates per genome, which was still too many to follow up using gene synthesis and cloning. To further winnow the possible candidates per genome, two additional criteria were introduced: a legitimate Shine–Dalgarno sequence and a predicted transmembrane domain (TMD). This narrowed the total number of candidates to 1–5 per genome, a manageable number. We analyzed 224 genomes (complete or partial) and 158 lysis gene candidates satisfying all three criteria were identified (Supplementary Data 1). For all 158 candidates, we synthesized genes and cloned them into an arabinose inducible plasmid vector. The candidates were then tested for function in *E. coli* by streaking on inducing agar, scoring as positive if the colonies showed growth inhibition when compared to the empty vector control. To test the possibility of *sgl*s falling outside of our initial criteria we also synthesized and tested an additional 135 candidates that either lacked a ribosomal binding site (RBS), fell below the 25-codon-length cutoff, or had less than an ideal TMHMM score. We found two functional genes that had no recognizable RBS and no predicted TMD (Supplementary Data 1 and Supplementary Fig. 2), five functional genes with no RBS but with a predicted TMD, three functional genes with a RBS but no predicted TMD (Supplementary Fig. 2), and one functional gene below the 25 codons in length with no RBS. All 33 candidates that scored positive on inducing agar were then tested for function by induction in liquid batch culture (Supplementary Fig. 3). There were only seven that showed overt lysis, but the other 26 showed a detectable growth inhibition phenotype; for simplicity, all of these will be referred to as functional. Considering that the natural hosts of these phages are not known, the failure to retain robust lytic function when expressed in the *E. coli* context is not surprising. Importantly, 178 out of 293 candidates, each with a predicted TMD, showed no effect on plating defect on inducing medium. This indicates that the simple existence of a TMD in a leviviral protein does not necessarily impose a growth inhibition or lytic phenotype under our conditions.

**Fig. 1:**
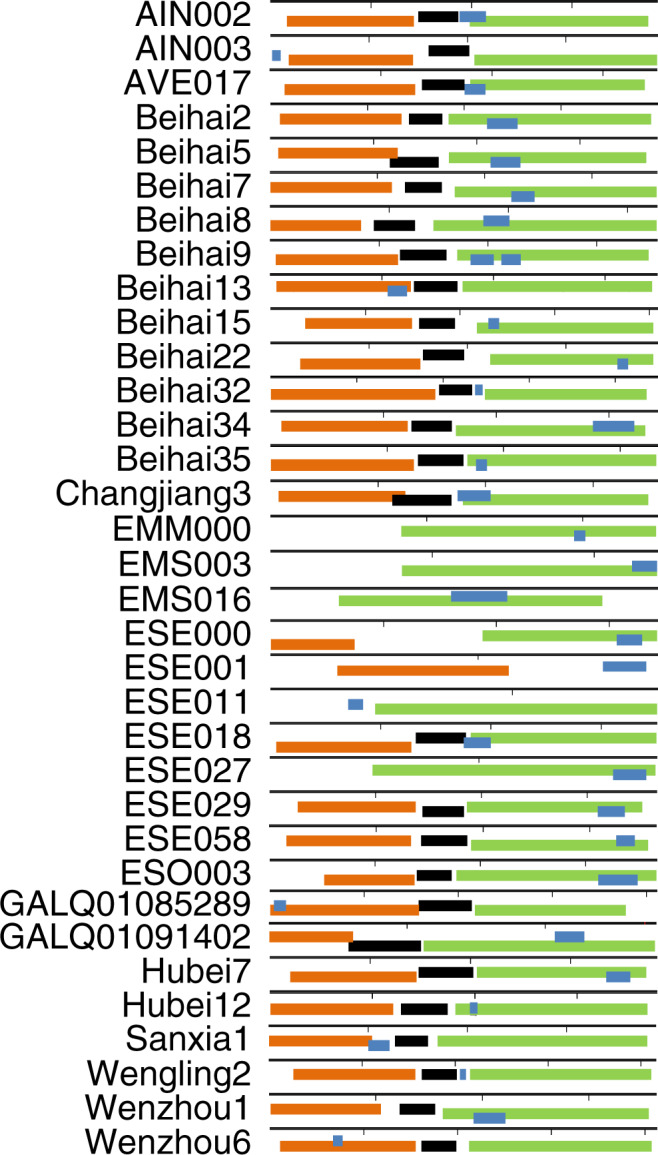
Genome organization of *sgl*s discovered in this study. The genome organization of leviviruses with functional *sgl*s are shown and the three core genes *mat*, *coat*, and *rep* are colored as orange, black, and green, respectively. The length of complete or partial genome is represented by a horizontal black bar above the genes with each perpendicular interval equal to 1 kb. The *sgl*s (blue) are shown relative to the reading frames of other genes (relative reading frames are represented by different horizontal levels). Beihai9 has two different *sgl*s in the genome and both are embedded within the *rep* gene.

Mapping these 33 *sgl*s on their respective genomes revealed that these genes have evolved in different locations, thus creating distinct levivirus genetic architectures (Fig. 1). Almost all of the Sgls discovered in this study share no significant (>50%) sequence similarity with each other or to any of the previously known eight Sgls from classic ssRNA phages (Supplementary Fig. 4). More strikingly, a BLAST of both the classic and new Sgls against the ~16,000 new leviviral genomes had only a few hits, thus highlighting the diversity of Sgls (Supplementary Tables 2 and 3).

### A majority of functional and annotated candidate *sgl*s are embedded in *rep*

**Fig. 2:**
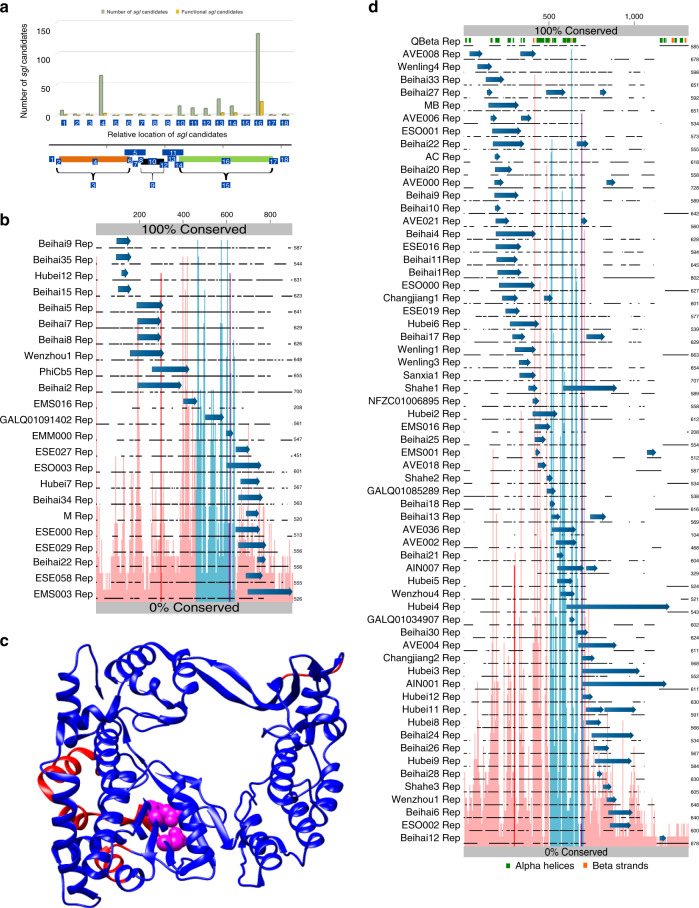
Genomic hot spots for *sgl* evolution. **a** Relative location of *sgl*-candidates within a canonical ssRNA phage genome architecture. Numbers 1 through 18 refer to the sub-locations within the genome where *sgl*-candidates could potentially be found. The bars in the graph show the total number of *sgl*-candidates (light green) and functional *sgl*s (orange) per sub-location. **b** An alignment of Rep sequences from phage genomes that have *rep*-embedded functional *sgl*s. The regions of sequence similarity are shown as lines and the gaps are shown as breaks in the lines. The location of the *sgl* in the context of replicase primary structure is shown as a blue arrow above the line. Sequence conservation among the Rep sequences is shown as pink bars (0% at the bottom gray bar, 100% conservation top gray bar). The bars corresponding to conserved motifs are colored as follows GPGA = red; motifs A–E = light blue; FRESCG = purple. **c** The relative location of functional *sgl*s mapped on to the crystal structure of Qβ RNA-Dependent RNA polymerase (RdRp) beta subunit (PDB:4R71). The structural elements that have tolerated evolution of embedded *sgl*s are highlighted in blue. The catalytically important residues are shown as magenta spheres and the regions without any functional *sgl*s are highlighted red. **d** An alignment of Rep sequences from phage genomes that have *rep*-embedded non-functional *sgl* candidates with Qβ Rep as a reference at the top. Annotations are the same as in **b**, except the secondary structure elements (alpha helices = green rectangles; beta strands = orange rectangles) are annotated above the Qβ Rep.

To understand the genetic context of *sgl* reading frames, each *sgl* candidate was further binned into 18 possible genetic contexts with reference to the core leviviral genomic structure (Fig. 2a). An analysis of the genomic context of all 293 *sgl* candidates revealed that most (195) were completely embedded in the two largest genes (64 in *mat* (region 4) and 131 in *rep* (region 16)). In contrast, only 15 candidates were completely embedded within the *coat* (region 10). Moreover, of the 33 candidates that showed activity in *E. coli*, more than 50% (21) are completely embedded within the *rep* gene in the +1-reading frame. This apparent bias could not be explained by differences in codon usage between *mat* and *rep* genes in genomes with and without *rep*-embedded *sgl*s (Supplementary Fig. 5). To better understand this bias towards evolving *sgl*s within *rep*, we generated an alignment of full or near-full length Rep primary structures from the genomes with *rep*-embedded *sgl* genes (Fig. 2b and Supplementary Fig. 6). The corresponding *sgls* (blue arrows) were then mapped onto the regions in the Rep primary structure that shared the same codon space. At first glance, this mapping revealed that *rep*-embedded *sgl* genes had evolved throughout the Rep primary structure. But upon closer inspection, it is clear that most *sgl*s are in less conserved regions of *rep* and can be broadly localized to two clusters; one in the N-terminal half and the other in the C-terminal half of *rep*. Furthermore, nearly half of the candidates in the N-terminal cluster overlap a conserved GPGA motif in Rep. This motif is conserved in the Rep primary structure and it is in the linker region connecting a pair of helices that are part of the finger domain of Rep, which has the hand overall structure^22^ (Fig. 2b, c). The *sgl* candidates that were judged to be functional and have evolved in the C-terminal half of Rep are also near a conserved motif GXFRESCG, which is part of the conserved motif E in RdRps^23^. Mutations in the first Gly in GXFRESCG motif are lethal to the function of replicase, which points to the high degree of constraints on base composition in the corresponding regions of the RNA. Only two of the *sgl*s that showed function in *E. coli* (GLAQ01091402 and EMM000) have evolved within the highly conserved central region (motifs A–E) of Rep. To gain insights into the effect *sgl* reading frames have on the tertiary structure of Rep, we mapped the regions that gave rise to Sgls onto the corresponding amino acids in the crystal structure of Qβ Rep. Strikingly, almost all regions of the structure have evolved Sgls, including the core catalytic regions (Fig. 2c). We wondered if this broad clustering of *sgl*s within the Rep primary structure was also observed for the candidates that scored negative in our initial screen in *E. coli*. We aligned the respective Rep sequences and then mapped the failed *sgl* candidates on the alignment (Fig. 2d). Surprisingly, most of the candidates still mapped to the less conserved N- and C-termini of Rep. Only a few of them are fully embedded in the central highly conserved region.

### ssRNA phage genomes with more than one *sgl* per genome

**Fig. 3:**
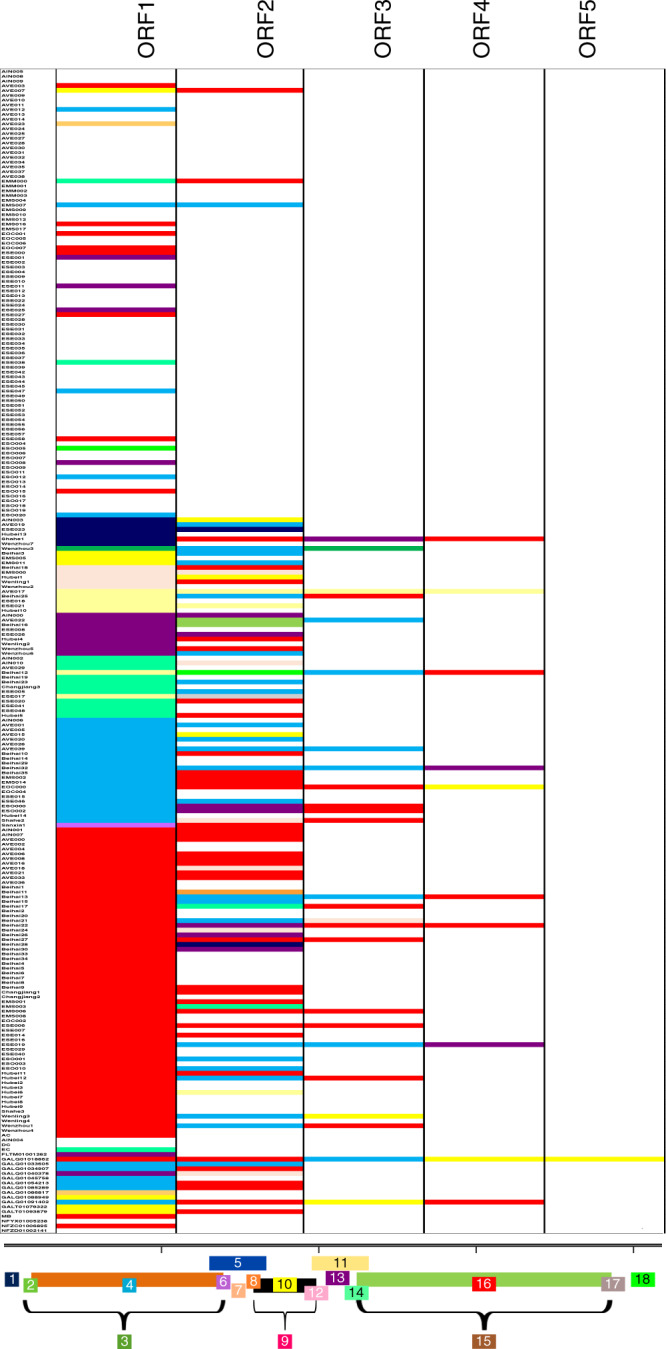
Relative location of *sgl*-candidate ORFs. The number and relative location of *sgl*-candidate ORFs that were synthesized and tested per genome. The relative genomic locations (1–18) are shown on the canonical ssRNA phage genome and color coded as follows: 1 (navy blue), 2 (lime green), 3 (army green), 4 (teal blue), 5 (royal blue), 6 (orchid purple), 7 (tan), 8 (orange), 9 (bright pink), 10 (bright yellow), 11 (pale yellow), 12 (rose pink), 13 (dark purple), 14 (seafoam green), 15 (brown), 16 (red), 17 (gray), 18 (neon green).

**Fig. 4:**
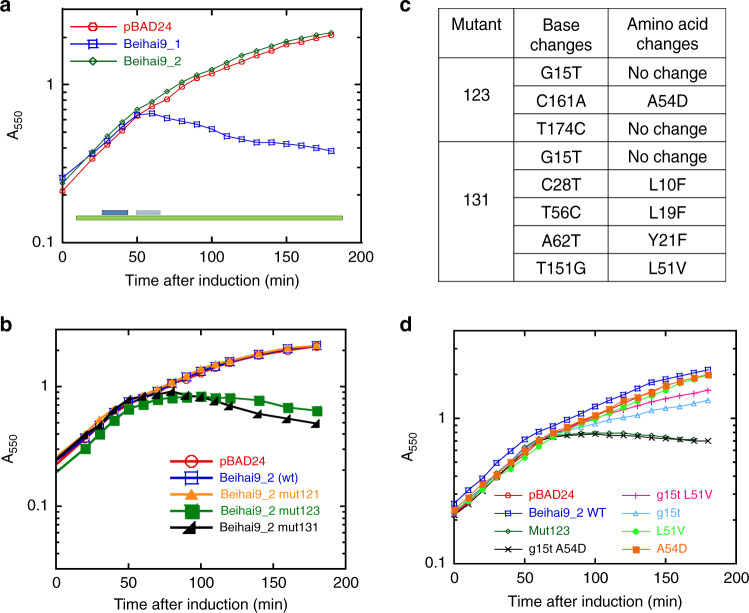
ssRNA phage with two functional lysis genes. **a** Lysis profiles of two *sgl* candidates (Beihai9_1 and Beihai9_2); pBAD24 (red open circle), Beihai9_1 (blue open squares), Beihai9_2 (green open diamond).The genomic context of *sgl*s is shown relative to the *rep* gene (green bar) with the functional candidate (Beihai9_1) shown as dark blue and the non-functional candidate as light blue. **b** The lysis profiles of Beihai9_2 gain-of-function mutants 123 and 131; pBAD24 (red open circle), Beihai9_2 (blue open square), Beihai9_2 mut121 (light orange-filled triangle), Beihai9_2 mut123 (green-filled square), Beihai9_2 mut131 (black-filled right triangle). **c** Table summarizing the base and amino acid changes of the mutants in **b**. **d** The lysis profiles of Beihai9_2 of single and double mutant constructs; pBAD24 (red open circle), Beihai9_2 (blue open squares), Beihai9_2 mut123 (green open diamond), Beihai9_2 double mutant g15t A54D (black cross), Beihai9_2 double mutant g15t L51V (pink plus), Beihai9_2 g15t (light blue open triangle), Beihai9_2 L51V (light green-filled circle), and Beihai9_2 A54D (orange-filled squares). The lysis profiles shown here are representative of three biological replicates.

Of the 293 *sgl* candidates cloned and tested from 244 partial or near-complete genomes, only 168 genomes had *sgl* candidates, with some having up to five candidates per genome (Fig. 3 and Supplementary Data 1). Eighty-nine genomes had two candidates, 25 had three candidates, 10 had four candidates, and one had five candidates. However, none of them had two *sgl*s that exhibited function in *E. coli*. We reasoned that some ssRNA phages in their natural environments might be capable of infecting two or more evolutionarily distant hosts and thus might require multiple *sgl*s. For example, phage PRR1 infects both *E. coli* and *Pseudomonas* carrying the RR1 multiple-drug resistance plasmid (Supplementary Table 1). To test this hypothesis, we focused on 20 genomes that had at least two *sgl*-candidates within the *rep* gene. The Beihai9 genome had two candidates, with only one candidate (Beihai9_1) exhibiting function in *E. coli* in our initial screen (Fig. 4a). We sought to evolve Beihai9_2 through directed-evolution to gain lytic activity in *E. coli*. We constructed a PCR-mutagenized Beihair9_2 plasmid library consisting of ~5000 clones and used the plasmid release technique^10^ to enrich for mutant clones that now caused lysis. After two rounds of enrichment, 20 clones were tested, revealing two clones (123 and 131) that caused growth inhibition on inducer plates; both caused lysis after induction in liquid culture (Fig. 4b). Sequencing the two clones revealed that there were multiple mutations in both, with clones 123 and 131 having 3 and 5 single base changes, respectively (Fig. 4c). Reconstructing all eight of the single base changes separately in the parental plasmid did not restore the lytic phenotype. However, one set of double mutants (g15t and A52D) restored the lysis phenotype (Fig. 4d). Interestingly, the double mutant consisted of a silent G → T base change, which suggests that RNA structure might play a role in expression of the Sgl from the plasmid. Nevertheless, the results show that leviviruses are capable of carrying more than one lysis gene or an easily adaptable cryptic gene in addition to the main *sgl*.

### Evolution of Sgls in closely related ssRNA phages

**Fig. 5:**
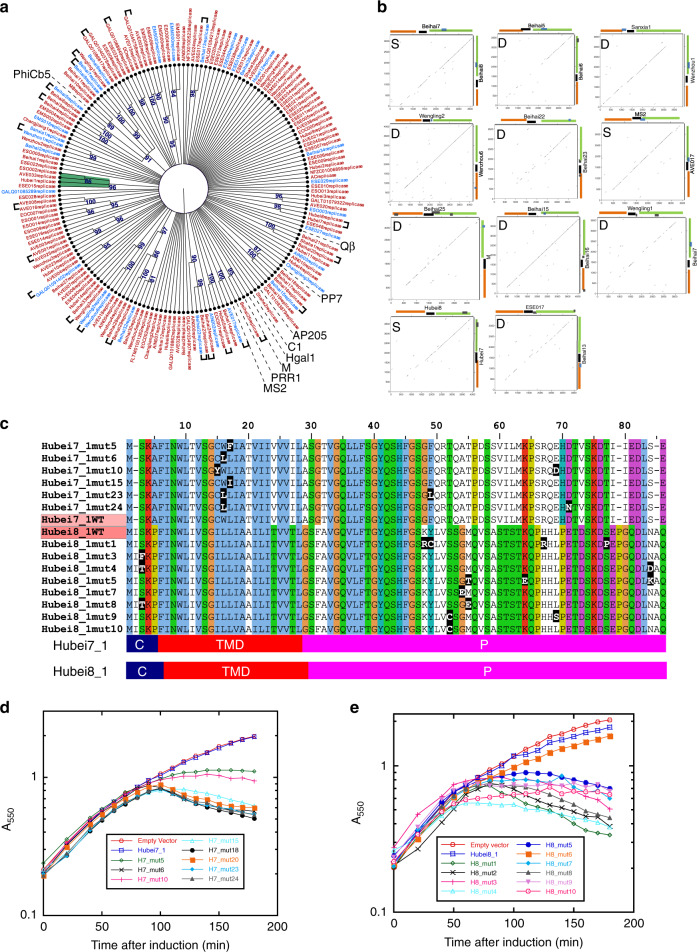
Evolution of Sgls in closely related ssRNA phages. **a** Cladogram of ssRNA phages based on the corresponding Rep primary structures. Nodes with bootstrap values >80 (resampling *n* = 1000) are shown and nodes with at least two levels are grouped as clades and are highlighted in eight different colors. The genomes with *sgl*s exhibiting function are colored blue, the *sgl*s from the previously characterized ssRNA phages are colored black, and candidates that tested as non-functional are colored red. Closely related phages are indicated by brackets. **b** Nucleotide dot plots of full length contigs of the closely related ssRNA phage genomes (*n* = 11 genome pairs). Each dot plot compares two genomes and the dot plots are categorized as S (similar) and D (different) based on the relative genomic context of *sgls*. **c** An alignment of Hubei7_1 and Hubei8_1 wild-type and gain-of-function mutants. The amino acids in the alignment are highlighted according to clustalX and the missense changes found in gain-of-function mutants are highlighted as white letters on black background. The respective predicted membrane topologies are shown below the alignment as bar diagrams with different colors representing different predicted subcellular localizations. Blue (cytoplasmic, C), red (transmembrane domain, TMD), and purple (periplasmic, P). **d** The lysis profiles of gain-of-function Hubei7 mutants; pBAD24 (red open circle), Hubei7_1 (blue open squares), H7_mut5 (green open diamond), H7_mut6 (black cross), H7_mut10 (pink plus), H7_mut15 (light blue open triangle). H7_mut18 (black-filled circle), H7_mut20 (orange-filled square), H7_mut23 (blue-filled diamond), H7_mut24 (light gray-filled triangle). The mutants H7_mut6, mut18, and mut20 have the same change (W15L). **e** The lysis profiles of gain-of-function Hubei8 mutants; pBAD24 (red open circle), Hubei8_1 (blue open squares), H8_mut1 (green open diamond), H8_mut2 (black cross), H8_mut3 (pink plus), H8_mut4 (light blue open triangle). H8_mut5 (blue-filled circle), H8_mut6 (orange-filled square), H8_mut7 (blue-filled diamond), H8_mut8 (light gray-filled triangle), H8_mut9 (light pink-filled inverted triangle), H8_mut10 (pink circled dot). H8_mut1 and mut2 are siblings and H8_mut6 (S53P and S58P) is non-functional. The lysis profiles shown in this figure are representative of three biological replicates.

To gain a better understanding of the evolution of *sgl*s in closely related ssRNA phages, we constructed cladograms based on the full or near-full length Rep or Mat sequences (Fig. 5a and Supplementary Fig. 7). Only the nodes with bootstrap values >80 were further analyzed. Based on this cutoff, 133 of the 174 Rep sequences considered could not be grouped into clades but the remaining 41 Rep sequences could be grouped into 8 clades. Of the clades that could be formed, the largest had 12 members and the smallest had three (Fig. 5a). In Fig. 5a, we highlighted the phage names with either blue (Sgls discovered in this study) or black (classic Sgls from Supplementary Table 1). To analyze the evolution of *sgl*s within closely related phages, we restricted our analyses mainly to nodes with bootstrap values >98, but exceptions were made if one of the phages had a functional Sgl (blue or black highlight). A total of 44 Rep sequences fit the above criteria and their corresponding genomes were compared as multiple sequence dot plots to find similarity across the length of the genome (Supplementary Fig. 8). These analyses showed that 11 of the 44 genomes have varying degrees of nucleotide similarity across the genome (Fig. 5b**)**. Most strikingly, at the nucleotide level the regions corresponding to the *mat* gene (5′-half of the genome) were more divergent than the 3′-half of the genome, which suggests that changes in host-specificity determinant (Mat) occur more frequently than in either *coat* or *rep*. We wondered if changes in the *mat* gene correlated with the changes in the corresponding *sgl*. Based on the genomic context of the *sgl*s, the 11 pairs could be separated into two sub-classes; the D-class (different) consisting of genome pairs where the *sgl* or *sgl*-candidates arose in different genomic locations and the S-class (similar) where the genetic context remained the same but the sequence has diverged (Fig. 5b). Eight genome pairs belonged to the D-class, of which two pairs (Sanxia1/Wenzhou1 and Wengling2/Wenzhou6) had functional *sgl*s that had evolved in different locations within the respective genomes. Of the remaining six pairs, only one of the phages in each pair had a functional *sgl* while the corresponding sequence in the other phage diverged away, as a new *sgl* evolved in a different location. The evidence for such divergence is found in the sequence of the corresponding phage pairs. In the case of the phage M/Beihai25 pair, *lys*^*M*^ the *sgl* of phage M is embedded in the 3′-half of *rep* gene, but the corresponding region in Beihai25 lacks an ORF. Nevertheless, the remnants of the *lys*^*M*^ gene can still be found in the Beihai25 genome (Fig. 5b and Supplementary Fig. 9). Similar observations of gene loss can be made in Beihai15/Beihai16. Among the three S-class phage pairs, only MS2/AVE017 have *sgl*s that tested functional; even here, the Sgls share only 38% amino acid sequence identity. Interestingly, the S-class pair Hubei7/Hubei8 have *sgl*s that failed the initial function test in *E. coli*. The putative Sgls share ~47% sequence identity, with the predicted TMDs differing at eight positions and with highly divergent periplasmic domains (Fig. 5c). Taken together, the conservation of the elements that make up a gene, such as Shine–Dalgarno and start/stop codons, similar gene length, conservation of predicted TMDs, and sequence identity over the length of the protein suggests that these ORFs are functionally relevant in the native hosts. To test this hypothesis, we used the plasmid release method to evolve variants of *sgl*^*Hubei7_1*^ and *sgl*^*Hubei8_1*^ capable of lysing *E. coli* (Fig. 5c). Of the 24 *sgl*^*Hubei7_1*^ clones screened after two rounds of plasmid release, six unique mutants were isolated. All six unique gain-of-function mutants of *sgl*^*Hubei7_1*^ have at least one mutation localized around Trp15, with three mutants replacing Trp with Leu, while the other three mutants have missense changes at position 14 or 16 (Fig. 5c). Phenotypically, the three single missense mutants (mut5 (L16I), mut6 (W15L), and mut15 (L16F)) display two different lysis profiles, with the former two causing a rapid drop in optical density and the latter displaying a more gradual drop (Fig. 5d). On the other hand, mutations in the gain-of-function mutants of *sgl*^*Hubei8_1*^ are predominantly localized in the highly divergent predicted periplasmic domain (Fig. 5c, e). In addition, a missense change near the N-terminus (S3F) also appears to be sufficient to elicit lysis in *E. coli* (Fig. 5c). Thus, it is clear from the above experiments that both *sgl*^*Hubei7_1*^ and *sgl*^*Hubei8_1*^ are functionally relevant *sgl*s that have diverged away from each other, presumably after the phage adapted to infect a different host species. Moreover, this result suggests that many, if not most, of our candidate Sgls that failed the function test in *E. coli* are likely to be real Sgls in the context of the most common host.

### De novo gene evolution in genomes sourced from geographically proximal regions

**Fig. 6:**
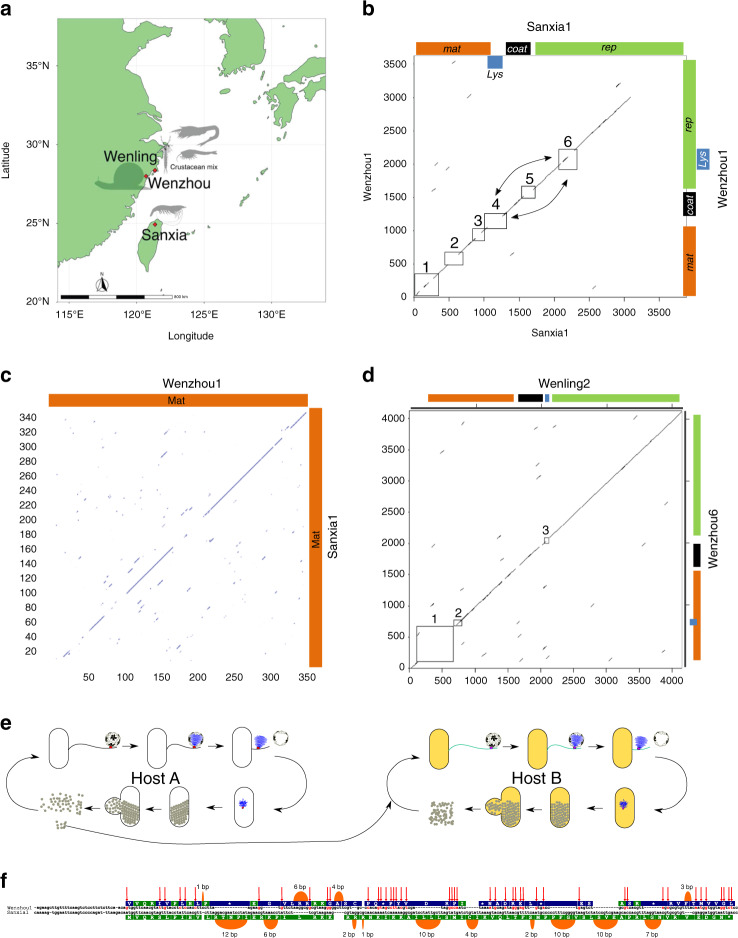
De novo gene evolution in ssRNA phages sourced from relatively close geographic locations. **a** Geographic locations and the invertebrate animal sources for the transcriptomes that contained the genomes of Wenzhou1, Wenzhou6, Wengling2, and Sanxia1. Silhouette images were obtained from phylopic.org, courtesy of Christoph Schomburg (shrimp), Joanna Wolfe and T. Michael Keesey (pan-Crustacea), and Scott Hartman (snail) under CC0 1.0 (https://creativecommons.org/publicdomain/zero/1.0/). **b** Nucleotide dot plot between Sanxia1 and Wenzhou1 genomes. The gaps in the first ~3000 bases of the alignment are boxed and numbered. Bi-directional arrows indicate the shift in the location of the *sgl*s. **c** Dot plot of the maturation protein primary structures of Sanxia1 and Wenzhou1. **d** Similar to **b** but Wenzhou6 vs Wenling2 comparison. **e** Model for de novo lysis gene evolution after changing pili specificity and presumably the host. **f** Sequence alignment of the region represented in box 4 of panel **b** with base changes highlighted red and indicated with red arrows. The indels are shown as orange loops with the size of the indel indicated above the loops. The translated reading frames are shown above (Wenzhou1) or below (Sanxia1) the nucleotide sequence and the amino acids shared between Sgl^Sanxia1^ and Wenzhou1 reading frame are colored green.

The disparate *sgl*s discovered in leviviruses sourced from global metatranscriptomics suggests that some arose through de novo gene evolution events. To understand such events in the context of both the geographical location and the source of the metatranscriptomes, we focused on the genome pairs Sanxia1/Wenzhou1 and Wengling2/Wenzhou6 which show extensive sequence similarity but have evolved functional *sgl* genes in different places (Fig. 5b). These four genomes were discovered from the metatranscriptomes of invertebrate animals sourced from regions within a ~200 km radius near the East China Sea (Fig. 6a). Sanxia1 was sourced from a shrimp on the island of Taiwan, while Wenzhou1/Wenzhou6 and Wenling2 came from an apple snail from the city of Wenzhou and crustacean mix from the city of Wenling, respectively. The dot plot of the genomes Sanxia1 and Wenzhou1 shows overall sequence similarity in the first ~3000 bases with only six distinct regions (boxed and numbered 1–6) lacking similarity (Fig. 6b). Mapping of the respective genetic architectures on the dot plot revealed that *sgl*s in Sanxia1 and Wenzhou1 arose in regions 4 and 6, respectively. Of the remaining four boxed regions, boxes 1, 2, and 3 encompass the region encoding the Mat protein, which provides pilin-specificity and attachment to the host pilus. A protein dot plot of the Mat proteins from Sanxia1 and Wenzhou1 showed that the N-terminal half or the putative pilin binding half of the proteins diverged from each other (Fig. 6c). Similarly, the *sgl*s in other two highly similar phages, Wenling2 and Wenzhou6, arose in places that diverged away from each other and the genomes also differ in the regions encoding N-terminal half of Mat proteins (Fig. 6d and Supplementary Fig. 10). Interestingly, the observation that genome differences also map to *mat* genes suggests that changes in the Mat protein alter the pili-specificity and confer the ability to infect a different and possibly distant host species. This adaptation to evolutionarily distant hosts likely renders the existing *sgl* useless and drives the evolutionary pressure to evolve a *sgl* de novo (Fig. 6e). To understand the specific base changes that led to the birth of a *sgl* gene in Sanxia1, we compared the nucleotide sequence of *sgl*^*Sanxia1*^ (bases 968..1228) to the corresponding region in Wenzhou1 (bases 975..1183). The alignment showed that *sgl*^*Sanxia1*^ arose de novo from a stretch of bases without any recognizable ORF in any of the three reading frames (Fig. 6f). Interestingly, one of the reading frames has 11 aa that are conserved in Sgl^Sanxia1^; moreover, to give rise to a *sgl*, the reading frame underwent 45 single base changes and 14 separate indels ranging in size from 1 to 12 bp (Fig. 6f).

## Discussion

Recent advances in next-generation sequencing technologies and directed searches for RNA viruses have either intentionally or unintentionally facilitated the discovery of thousands of leviviruses^18–21^. However, almost all of the ssRNA phage genomes sourced from metatranscriptomes lack an annotated lysis gene or *sgl*, which is not surprising because most of the known Sgls are small, are encoded in alternate reading frames of essential genes, and lack sequence similarity to other Sgls. To tackle the missing gene problem, we took the simple approach of identifying ORFs in 244 genomes, cloning the putative *sgl* candidates on inducible plasmids, and testing them for lytic or growth inhibitory effect in *E. coli*. This led to the identification of 35 unique Sgls (33 in the initial screen plus two evolved Sgls) exhibiting activity in *E. coli*, each potentially representing a distinct mechanism to effect host cell lysis. Moreover, a BLAST search with the expanded pool of Sgls against the recently deposited tens of thousands of leviviruses returned only a handful of hits, which suggests that Sgls are extremely diverse and remain vastly untapped as a source for peptides that attack essential cellular functions; i.e., for protein antibiotics^24,25^.

One lysis gene per phage genome has been the paradigm for small lytic phages (microviruses and leviviruses) since the late 1970s, when the first genomes, φX174 and MS2, respectively, were sequenced^26,27^. The results presented in this study show that a ssRNA phage could potentially have two or more lysis genes at different stages of gene evolution. One reasonable hypothesis for multiple *sgl*s per genome is that leviviruses are capable of infecting different host species, mainly due to their retractable pilus tropism, rather than specificity to host surface receptors. Therefore, the selective pressures to maintain more than one *sgl* per genome may arise in an environment of near constant passage of the virus from one species to the other. In the absence or loss of such pressure, the other *sgl*s diverge in their sequence to become cryptic and ultimately lose the basic features of a gene. The evolution of disparate *sgl*s in closely related phages suggests rapid gene evolution, but the timescale and frequency of such events is not known. By mining the ssRNA phage sequence space and doing in vitro reconstruction experiments, insights into such events and the evolutionary steps that turn a stretch of nucleic acids into a new gene with a new function could be obtained.

Another wide-ranging impact of this study comes from the observation that a large proportion of *sgl*s have evolved completely within the *rep* gene, specifically in the +1 reading frame. Even though *mat* and *rep* genes are similarly sized, a disproportionate number (22 of 35) of *sgl*s or *sgl*-candidates were found embedded within the *rep* gene. This disparity could not be explained by differences in sequence attributes such as codon usage. Interestingly, most of the shared sequence space in the *rep* gene encodes highly divergent regions of Rep, with the near universally conserved catalytic regions of Rep (motifs A and C) harboring only a few *sgl*s. Therefore, it is reasonable to hypothesize that the highly divergent regions are more permissible for exploration of codon-space, especially in alternate reading frames, and thus serve as hotspots for *sgl* evolution. Moreover, Rep relies on host proteins such as ribosomal protein S1, EF-Tu, EF-Ts, and Hfq (for Qβ) to form a replication-competent holoenzyme^28–31^. These host proteins could also serve as a structural scaffold to attain the optimal conformation of RdRp. Thus, this added flexibility allows the *rep* gene to explore sequence space much more than *mat* and *coat*. Evolutionarily, the leviviruses are thought to be ancestors of capsid-less eukaryotic viruses such as mitoviruses and narnaviruses, which are found in fungi as naked RNA replicons in the mitochondria and the cytosol, respectively^32^. It has been speculated that RdRp genes were passed on through modular gene exchange to various lineages of eukaryotic RNA viruses. Hence, it might be worthwhile to investigate the RdRp genes from other RNA viruses for the propensity to carry small embedded genes that may play a critical role in pathogenesis.

By analyzing a relatively minuscule sample of the total leviviral universe or leviverse, we have uncovered an incredible diversity of small peptides that carry out a critical function in the life cycle of RNA viruses. Our results motivate further research into exploiting these peptides for identifying targets for antibiotic development, to uncover small genes and their biological functions in RNA viruses of higher eukaryotes, and provide a good model system for studying de novo gene evolution and origins.

## Methods

### Bacterial strains, plasmids, primers, and growth conditions

The plasmids used in this study are listed in Supplementary Data 1 and Supplementary Table 4. Bacterial strains used in this work are XL1Blue (Stratagene) and DH5α (ThermoFisher Scientific). Primers and synthetic DNA (g-blocks) are listed in Supplementary Data 2. Cultures were grown with aeration at 37 °C in lysogeny broth (LB) supplemented with ampicillin (100 μg mL^−1^) and L-arabinose (0.4% w/v) when indicated.

The *sgl* candidate ORFs (with or without codon optimization for *E. coli*) flanked by restriction sites for EcoRI and XhoI/HindIII upstream and downstream, respectively, were synthesized as g-blocks (IDT). Each g-block contained two to five *sgl* candidates with each candidate flanked by the above restriction sites to facilitate cloning. The g-blocks were digested with EcoRI/HindIII or EcoRI/XhoI enzymes and cloned into similarly digested pBAD24 (empty vector) or pKC3, respectively^16^. The clones were verified by Sanger sequencing (EtonBiosciences) with primers KC30 or KC31.

Mutant alleles of *sgl*^*Beihai9_2*^ were constructed by site-directed mutagenesis on the plasmid pBAD24 Beihai9_2 using Phusion high-fidelity DNA polymerase and the primers KC737, KC738, KC739, KC740, KC741, KC742, KC745, KC746, KC747, KC748, KC749, KC750, KC751, KC752, KC753, KC754, KC755, and KC756.

### Chemical reagents and enzymes

All chemicals were purchased from Sigma-Aldrich unless otherwise stated. All enzymes and the associated buffers were purchased from New England Biolabs unless otherwise stated.

### Annotation of *sgl*-candidates

The ssRNA phage genomes were sourced from previously deposited and/or published datasets^18,19^; see Data Availability, below. The *sgl* candidates in these genomes were manually annotated using SnapGene (GSL Biotech LLC). All ORFs greater than 25 amino acids with either ATG or GTG or TTG start codons were selected, analyzed for a putative Shine–Dalgarno sequence, and the corresponding protein sequences were analyzed for predicted transmembrane domain (TMD) using TMHMM server, v.2.0 http://www.cbs.dtu.dk/services/TMHMM/. The ORFs that passed the above three criteria were synthesized, cloned, and assayed for lytic function in *E. coli* XL1Blue. One or more exceptions to the three criteria listed above were made while annotating some *sgl* candidates.

### Functional characterization of sgl-candidates

The transformants carrying the *sgl* plasmids were streaked on inducer (0.4% w/v L-arabinose) and non-inducer LB agar plates supplemented with ampicillin (100 μg mL^−1^) and incubated at 37 °C overnight. The clones that showed growth inhibition only on the inducer plates were scored as positive hits. The positive clones were further characterized by following their lysis profiles. Lysis profiles were obtained by taking 125 µL of overnight cultures and adding them into respective 250 mL culture flasks with 25 mL of LB supplemented with ampicillin (100 µg/mL). The flasks were incubated in a 37 °C water bath shaker and induced at A_550_ = 0.2 with arabinose. After induction, the optical density was determined at regular intervals and the data was plotted using Kaleidagraph version 4.03 (Synergy Software).

### Mutant library construction and plasmid release

The lysis gene candidates were mutagenized using the GeneMorph II Random Mutagenesis Kit (Agilent Technologies) as per manufacturer’s instructions using primers KC30 and KC31. To ensure high mutation rate, low initial template (1 ng/µL) and 35 cycles of amplification were used. The PCR product was digested with DpnI to remove template plasmid DNA and purified using PCR clean up kit (Qiagen). The purified mutant PCR product was digested with EcoRI and HindIII and ligated into similarly cut pBAD24 vector. The ligated product was then transformed into MAX Efficiency DH5α-T1^R^ competent cells (ThermoFisher Scientific). The mutation frequency was assessed by sequencing 10 colonies and the cloning process was repeated until desired number of colonies were obtained.

To pool the mutant library, 5 ml of LB was added per plate and the colonies were scraped off the plate using a sterile glass rod. The pooled cell suspension was diluted 10-fold and 125 µL of the diluted suspension was used to inoculate 25 mL LB media supplemented with ampicillin (100 µg/mL) in a 250 mL Erlenmeyer flask. Cultures were aerated at 37 °C in a water bath shaker (New Brunswick Scientific Gyrotory G76) until the O.D_550_ reached 0.2, at which point the cultures were induced with arabinose (0.4% w/v final concentration). Two hours post induction the cultures were harvested and centrifuged for 10 minutes at 10,000 × *g* at 4 °C. The supernatant was filtered through 0.22 µm syringe filter into a new 50 mL falcon tube. At this point 1/10 volume of 3 M Sodium acetate pH 5.2 and 1 volume of isopropanol was added to the filtrate, and then passed through a DNA spin column (Econospin^TM^). The columns were then washed with 2 mL of Qiagen PE wash buffer and bound DNA was eluted in 50 µL of sterile water. Then 5 µL of the eluted DNA was transformed back into DH5*α* T1^R^ cells and transformants were pooled and subjected to another round of plasmid release as described above. The whole process was repeated for a total of two to three rounds.

### Dot plots and multiple interrelated sequence doT (MIST) plots

Nucleotide dot plots were generated with dotmatcher^33^ with window size and threshold set at 40.0 and 50.0, respectively. MIST v3 was used to generate complete NxN plot of related genomes. The code for dotmatcher and MIST tools are available from CPT Galaxy Tools, which is available at 10.5281/zenodo.4048782.

### Multiple sequence alignments (MSA), pair-wise alignments, and phylogenetic analyses

The MSA, phylogenetic analyses of Mat and Rep primary structures, and pair-wise alignments of Sgls were done using CLC Genomics Workbench 8.0.1 (Qiagen). The MSAs were done with gap open and extension costs of 10.0 and 1.0, respectively. Circular cladograms were constructed using Neighbor-Joining method and bootstrap analyses with 1000 replicates.

### Codon usage (CU) analysis

CU was determined using Measure Independent of Length and Composition (MILC) method^34^. The CU analyses were done using an R package, coRdon, from Bioconductor^35^. A R-script was written to calculate and plot MILC values for individual *mat* and *rep* genes (Supplementary Software). The codon distribution was calculated from corresponding *mat* and *rep* genes and plotted on *X* and *Y* axis, respectively.

### Qβ replicase structural rendering

The relative locations of the functional-*sgl*s were rendered on the crystal structure of Qβ replicase beta subunit (PDB: 4R71) using the UCSF Chimera package^36^.

### Reporting summary

Further information on research design is available in the Nature Research Reporting Summary linked to this article.

## Supplementary information

Supplementary Information

Peer Review File

Description of Additional Supplementary Files

Supplementary Data 1

Supplementary Data 2

Supplementary Software

Reporting Summary

## Data Availability

All data generated and analyzed during the current study are available from the corresponding author upon reasonable request. The source data for Figs. 4a, b, d, 5d, e, and Supplementary Fig. 3a–d are available in the Source data file. The ssRNA phage genome sequences used in this study were sourced from previously deposited and/or published sources^18,19^. Of the 244 genomes, 96 have GenBank accession numbers (see Supplementary Data 1) and the rest are available at 10.1371/journal.pbio.1002409.s001. The structure of Qβ replicase beta subunit was obtained from Protein Data Bank (PDB:4R71). Source data are provided with this paper.

## Source data

Source Data

## Code availability

The code used in this study for MIST and dot plots is available from public repository at 10.5281/zenodo.4048782. The R scripts used for codon usage analysis and for generating Fig. 6a are provided as Supplementary Software.
