# Supplementary material for: Rapid de novo evolution of lysis genes in single-stranded RNA phages
Source: Nat Commun. 2020 Nov 26;11:6009. doi: 10.1038/s41467-020-19860-0 (PMC7693330; doi:10.1038/s41467-020-19860-0)
Supplement: Supplementary file 1 — Supplementary Information [file 41467_2020_19860_MOESM1_ESM.pdf]

Supplementary Information

for

Rapid evolution of lysis genes in single-stranded RNA phages.

Karthik R. Chamakura, Jennifer S. Tran, Chandler O'Leary, Hannah G. Lisciandro, Sophia F. Antillon, Kameron D. Garza, Elizabeth Tran, Lorna Min, and Ry Young.

This supplementary file includes:

Supplementary Tables 1-4

Supplementary Figures 1-10

Supplementary References.

Supplementary Table 1. Classic leviviruses and their Sgls

phage	pilus	host	*sgl* location	Sgl target	Sgl size (aa)
MS2	F	*E. coli*	*coat-rep* junction	L-target	75
Qβ	F	*E. coli*	= A_2	MurA	420
M	RIP69 (IncM)	*E. coli*	distal *rep*	MurJ	37
Hgal1	R27 (IncH)	*E. coli*	*coat-rep* junction	L-target	65
C-1	RA1(IncC)	*E. coli*	*coat-rep* junction	L-target	65
phiCB5	polar	*C. crescentus*	middle *rep*	?	136
PP7	polar type IV	*P. aeruginosa*	*coat-rep* junction	L-target	55
LeviOr01	polar type IV	*P. aeruginosa*	?	?	?
PRR1	R1822 (IncP-1)	*P. aeruginosa*	*coat-rep* junction	L-target	54
AP205	type IV twitching	*Acinetobacter*	5' of *mat*	L-target	35

Supplementary Table 2: Sgl BLAST hits against leviviral genomes from Starr, 2019

Query	Number of HSPs	Lowest E-value	Greatest identity %	Greatest positive %	Greatest HSP length
phiCb5_lys_protein	11	3E-28	76.19048	85.71429	378
Hubei7	24	1.03E-25	67.08861	73.41772	246
Qbeta_lys_protein	254	6.84E-18	72.72727	91.30435	1101
ESE029	28	6.87E-11	69.23077	92.30769	243
ESE000_1	10	0.026173	52.17391	65.21739	171
ESO003	8	0.047128	47.61905	61.90476	213
Beihai2	19	0.054513	66.66667	91.66667	171
Beihai7	6	0.175914	66.66667	83.33333	114
Wenzhou6	6	0.181112	60	80	81
Beihai35	4	0.208626	50	83.33333	99
GALQ010911402_4	21	0.237793	60	100	159
Beihai34_1	6	0.247428	44.44444	66.66667	180
MS2_lys_protein	12	0.268846	69.23077	92.30769	132
Beihai9_1	7	0.495581	69.23077	84.61538	117
Beihai9_2	14	0.547807	52.94118	71.42857	159
ESE001	26	0.598647	60	73.33333	162
AIN003_2	5	0.60525	66.66667	88.88889	63
GALQ01085289_1	20	0.662492	77.77778	77.77778	108
AIN002	4	0.688994	52.38095	66.66667	105
phageC-1_lys_prote	14	0.854713	64.28571	78.57143	117
phageM_lys_protei	3	0.861696	81.81818	81.81818	66
Wenzhou1	6	0.862423	47.61905	66.66667	177
Beihai13	15	0.995562	58.33333	83.33333	162
Beihai5	1	1.03793	45.83333	58.33333	72
Hubei12	1	1.08561	40.90909	72.72727	66
EMS016	10	1.39879	53.84615	84.61538	99
Changjiang3_1	8	1.40468	63.63636	90.90909	138
Hgal1_lys_protein	1	1.66418	36.36364	63.63636	66
ESE018	8	1.7311	57.14286	75	120
Beihai22	5	1.9689	46.66667	66.66667	105
AVE017	9	2.08092	57.14286	68	108
ESE058_1	3	2.53043	72.72727	84.61538	63
Sanxia1_ORF4	1	3.29002	35.71429	50	84
ESE027	3	4.3871	75	87.5	42
Beihai32	3	4.72631	53.84615	76.92308	45
Beihai8	2	5.2212	37.5	58.62069	87
Beihai15	5	5.79318	66.66667	66.66667	87
EMS003	1	6.55514	42.10526	57.89474	57
PP7_lys_protein	2	7.84658	50	66.66667	63
ESE011	2	7.86483	54.54545	72.72727	60
AP205_lys_protein	1	9.1479	52.94118	70.58824	51
Wenling2_1	0				
EMM000	0				
PRR1_lys_protein	0				

Supplementary Table 3: Sgl BLAST hits against leviviral genomes from Callanan, 2020 .

Query	Number of HSPs	Lowest E-value	Greatest identity %	Greatest positive %	Greatest HSP length
Qbeta_lys_protein	250	1.65E-51	64.54545	82.17822	969
ESE000_1	47	4.23E-49	92.20779	93.50649	231
ESE029	84	9.95E-40	75	81.81818	264
ESO003	35	3.67E-33	73.33333	82.35294	378
Beihai2	11	2.03E-11	57.14286	66.66667	207
ESE027	8	1.36E-07	61.70213	70.21277	156
Hubei7	81	5.63E-07	69.23077	76.92308	231
PRR1_lys_protein	2	0.005639	44.82759	62.06897	87
Beihai22	4	0.072905	52.94118	64.70588	108
phiCb5_lys_protein	3	0.08975	44.44444	64.44444	156
Beihai13	4	0.213556	38.46154	61.53846	138
GALQ010911402_4	16	0.291552	66.66667	75	153
Wenzhou1	12	0.308266	66.66667	83.33333	162
Beihai5	32	0.325821	71.42857	78.57143	96
phageC-1_lys_prot	7	0.356449	55	80	90
ESE018	23	0.373405	60.71429	66.66667	198
MS2_lys_protein	29	0.454107	75	91.66667	183
Beihai9_2	4	0.670468	47.61905	66.66667	120
Beihai35	8	0.750757	54.54545	90.90909	63
GALQ01085289_1	11	0.80948	56.25	80	120
Hgal1_lys_protein	22	0.89056	69.23077	83.33333	90
Sanxia1_ORF4	5	0.89946	45	80	87
phageM_lys_prote	1	1.11473	30	56.66667	90
Wenzhou6	3	1.25258	40.90909	58.62069	87
EMS016	7	1.39623	47.36842	73.91304	99
Beihai7	13	1.43868	50	68.75	147
Changjiang3_1	12	1.59443	56.25	75	117
ESE001	4	2.03044	53.84615	76.92308	114
Beihai9_1	6	2.11484	45	75	60
Beihai34_1	10	2.27915	34.61538	54	207
Beihai8	4	2.35592	57.89474	63.15789	87
Beihai15	9	2.53674	55.55556	77.77778	87
AIN002	4	2.78615	50	75	114
PP7_lys_protein	4	4.77686	50	66.66667	63
EMS003	2	4.99477	50	92.85714	60
AVE017	10	5.17106	64.70588	76.47059	90
Beihai32	3	7.12151	57.89474	89.47368	57
AP205_lys_protein	1	8.36542	27.27273	60.60606	99
AIN003_2	2	9.71555	52.63158	68.42105	57
Wenling2_1	0				
ESE058_1	0				
ESE011	0				
Hubei12	0				
EMM000	0				

Supplementary Table 4: Plasmids used in this study.

Plasmid construct	Relevant genotype	Origin	Source
pBAD24	*bla araC* P_{BAD}*::*	ColE1	Guzman, 1995
pKC3	*bla araC Para::lys-his6 lacZα*	ColE1	Chamakura, 2017
pBAD24 Beihai9_2	*bla araC* P_{BAD}*::Beihai9_2*	ColE1	This work
pBAD24 Beihai9_G15T	*bla araC* P_{BAD}*::Beihai9_G15T*	ColE1	This work
pBAD24 Beihai9_C161A	*bla araC* P_{BAD}*::Beihai9_C161A*	ColE1	This work
pBAD24 Beihai9_T174C	*bla araC* P_{BAD}*::Beihai9_T174C*	ColE1	This work
pBAD24 Beihai9_C28T	*bla araC* P_{BAD}*::Beihai9_C28T*	ColE1	This work
pBAD24 Beihai9_T56C	*bla araC* P_{BAD}*::Beihai9_T56C*	ColE1	This work
pBAD24 Beihai9_A62T	*bla araC* P_{BAD}*::Beihai9_A62T*	ColE1	This work
pBAD24 Beihai9_T151G	*bla araC* P_{BAD}*::Beihai9_T151G*	ColE1	This work
pBAD24 Beihai9_G14T	*bla araC* P_{BAD}*::Beihai9_G15T_C161A*	ColE1	This work
pBAD24 Beihai9_G14T	*bla araC* P_{BAD}*::Beihai9_G15T_T151G*	ColE1	This work

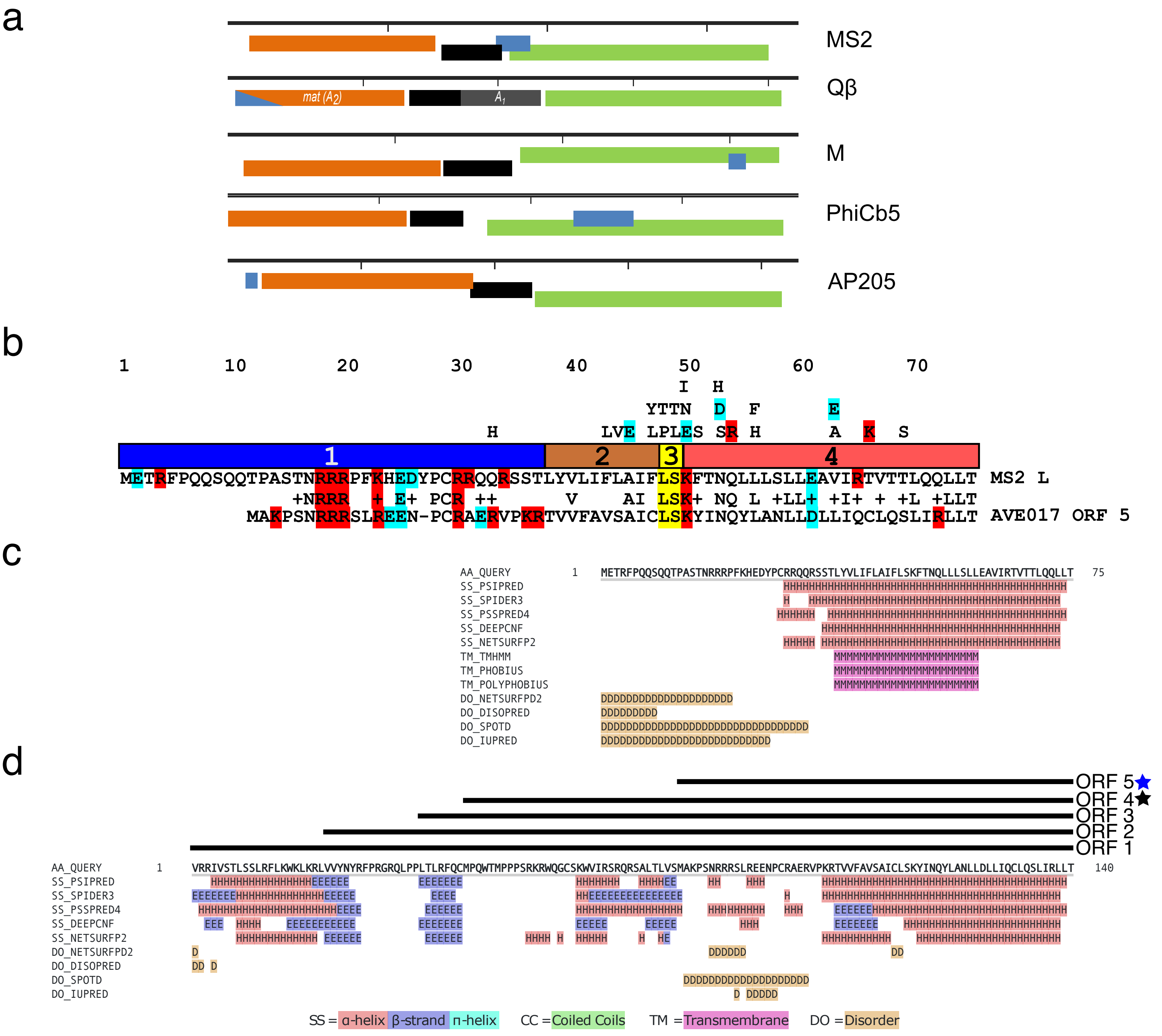

Supplementary Figure 1. Similarity between MS2 L and the Sgl^{AVE017}.

(**a**) Unique genetic architectures of classic leviviruses[3]. Color scheme and scale bar same as Figure 1. The Qβ coat read-through is colored gray (**b**) Primary structure alignment of MS2 L and ORF 5 of Sgl^{AVE017}. The 4-motif structure common to L-like proteins and non-functional mutants of L are shown above the L primary structure. (**c**) The Quick2D secondary structure predictions of MS2 L. (**d**) The Quick2D secondary structure predictions of the various ORFs of *sgl*^{AVE017}, the black horizontal bars represent the lengths of ORFs 1-5. ORF 4 (black star) is the annotated *sgl*^{4} and ORF 5 (blue star) is the only functional variant of *sgl*^{AVE017}.

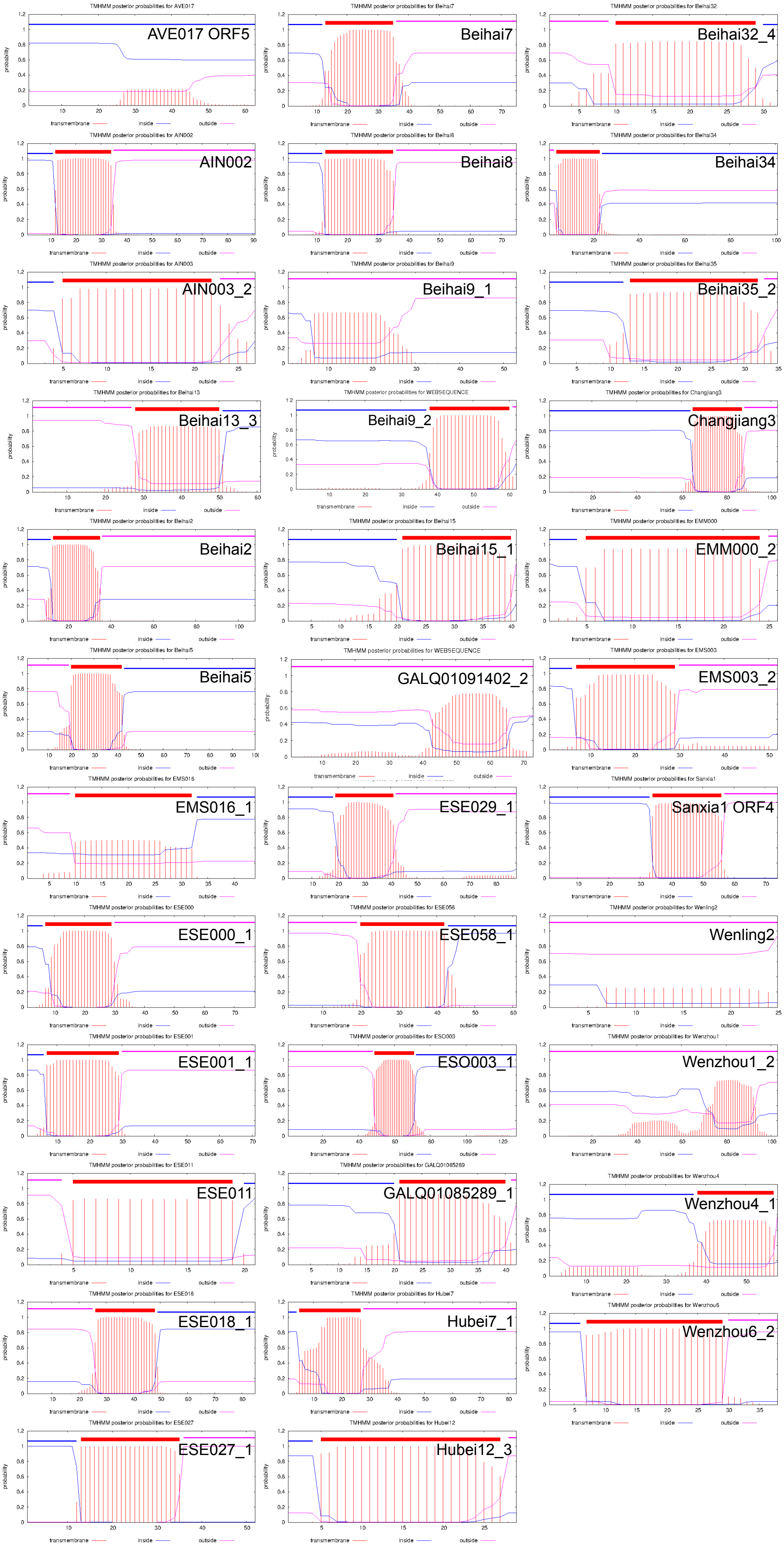

Supplementary Figure 2. Predicted Transmembrane domains of functional SgIs.
Putative membrane topology and predicted transmembrane domains of different functional SgI proteins. The TMHMM probability score is shown on the respective Y-axis and X-axis corresponds to amino acid length.

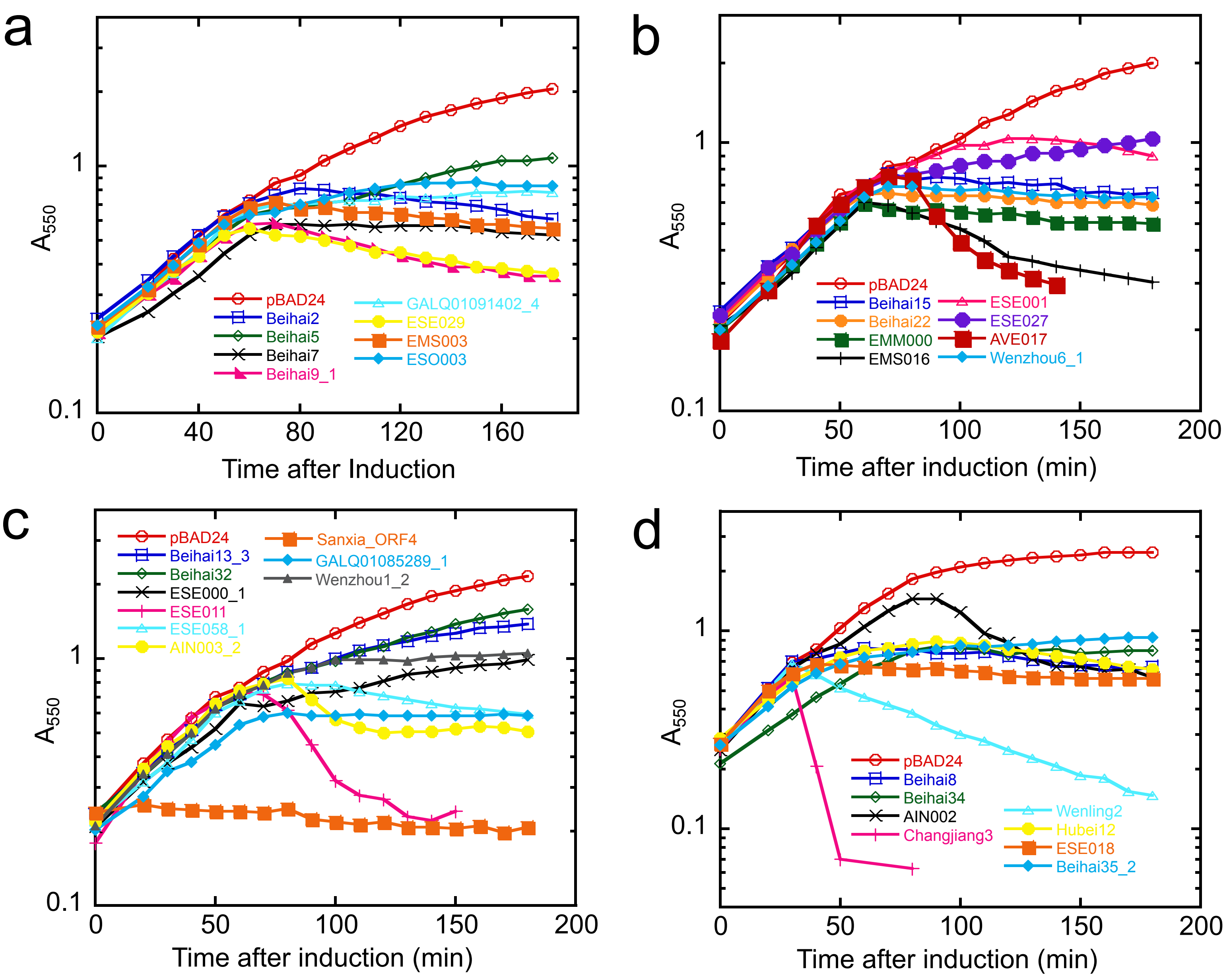

Supplementary Figure 3. Lysis profiles of functional SgIs discovered in this study.
(**a**) Lysis profiles of functional sgls: Beihai2 (blue open squares), Beihai5 (green open diamond), Beihai7 (black cross), Beihai9_1 (pink filled right triangle), GALQ01091402_4 (light blue open triangle), ESE029 (yellow filled circle), EMS003 (orange filled square), ESO003 (blue filled diamond), pBAD24 (red open circle). (**b**) Lysis profiles of Beihai15 (blue open squares), Beihai22 (light orange filled circle), EMM000 (dark green filled square), EMS016 (black cross), ESE001 (pink open triangle), ESE027 (purple filled circle), AVE017 (red filled square), Wenzhou6_1 (blue filled diamond). (**c**) Lysis profiles of Beihai13_3 (blue open squares), Beihai32 (green open diamond), ESE000_1 (black cross), ESE011 (pink cross), ESE058_1 (light blue open triangle), AIN003_2 (yellow filled circle), Sanxia_ORF4 (orange filled square), GALQ01085289_1 (blue filled diamond), Wenzhou1_2 (grey filled triangle). (**d**) Lysis profiles of Beihai8 (blue open squares), Beihai34 (green open diamond), AIN002 (black cross), Changjiang3 (pink cross), Wenling2 (light blue open triangle), Hubei12 (yellow filled circle), ESE018 (orange filled square), Beihai35_2 (blue filled diamond). The lysis profiles shown here are representative of three biological replicates.

	#	Beihai5	ESE027_1	Beihai15_1	EMS003_2	Beihai35_2	Beihai2	ESE018_1	Beihai13_3	Wenzhou1_2	Sanxia1_ORF4	EMS016_1	Beihai8	Beihai7	Beihai9_1	AIN003_2	Beihai32_4	ESE029_1	Beihai34_1	phageC1	Changjiang3_1	ESE058_1	ESE001_1
		1	2	3	4	5	6	7	8	9	10	11	12	13	14	15	16	17	18	19	20	21	22
Beihai5	1	100.00	17.00	2.50	6.00	3.64	2.24	4.92	2.00	3.12	5.45	5.00	7.00	2.00	6.00	4.00	2.00	1.48	1.52	0.64	4.20	5.00	2.78
ESE027_1	2	17	100.00	1.25	4.11	4.17	1.52	2.88	4.11	3.64	4.40	5.56	6.02	4.94	1.16	0.00	6.45	2.75	2.75	0.90	5.31	4.11	4.21
Beihai15_1	3	3	1	100.00	5.56	13.33	4.63	2.25	4.76	2.75	3.09	1.56	1.03	3.09	4.11	3.64	3.64	4.44	4.31	2.02	3.88	1.18	3.61
EMS003_2	4	6	3	4	100.00	7.94	7.02	1.08	3.95	5.31	2.97	4.11	7.59	6.33	2.50	1.69	1.69	3.12	2.52	2.75	6.14	7.46	4.55
Beihai35_2	5	4	3	6	5	100.00	4.55	3.49	5.19	1.87	2.30	3.64	2.30	3.45	3.12	4.08	8.51	2.22	2.56	3.30	5.83	1.30	2.74
Beihai2	6	3	2	5	8	5	100.00	5.17	3.88	5.76	2.88	2.52	3.79	3.79	3.67	2.68	3.42	11.02	4.20	1.83	7.91	3.10	5.31
ESE018_1	7	6	3	2	1	3	6	100.00	8.49	11.11	3.31	1.98	2.63	2.63	6.38	3.37	6.45	8.82	3.94	1.42	8.13	7.41	6.32
Beihai13_3	8	2	3	4	3	4	5	9	100.00	6.19	3.92	3.95	5.75	4.60	4.55	4.69	4.35	2.61	4.63	1.68	5.69	8.11	5.32
Wenzhou1_2	9	4	4	3	6	2	8	12	7	100.00	4.96	1.71	4.13	6.56	2.56	6.80	4.63	5.79	9.68	0.61	12.20	3.45	6.84
Sanxia1_ORF4	10	6	4	3	3	2	4	4	4	6	100.00	7.79	5.05	2.02	3.49	7.32	2.67	4.00	5.04	2.31	7.32	5.15	3.81
EMS016_1	11	5	4	1	3	2	3	2	3	2	6	100.00	6.33	5.06	4.69	5.66	6.00	2.88	3.48	3.00	3.48	6.49	7.41
Beihai8	12	7	5	1	6	2	5	3	5	5	5	5	100.00	45.45	6.25	6.49	3.90	8.55	3.97	1.50	5.74	8.97	4.72
Beihai7	13	2	4	3	5	3	5	3	4	8	2	4	35	100.00	7.29	8.97	5.19	4.27	3.97	2.29	4.92	7.69	4.72
Beihai9_1	14	6	1	3	2	2	4	6	4	3	3	3	6	7	100.00	9.38	4.69	5.50	4.17	2.75	3.12	2.13	9.59
AIN003_2	15	4	0	2	1	2	3	3	3	7	6	3	5	7	6	100.00	6.38	5.43	4.76	1.14	1.85	7.58	4.00
Beihai32_4	16	2	4	2	1	4	4	6	3	5	2	3	3	4	3	3	100.00	2.13	2.83	1.11	4.72	9.68	3.61
ESE029_1	17	2	3	4	3	2	13	9	3	7	5	3	10	5	6	5	2	100.00	8.87	2.05	9.73	2.75	3.67
Beihai34_1	18	2	3	5	3	3	6	5	5	12	6	4	5	5	5	5	3	11	100.00	2.52	11.90	5.26	3.15
phageC1	19	1	1	2	3	3	3	2	2	1	3	3	2	3	3	1	1	3	4	100.00	1.89	0.84	1.57
Changjiang3_1	20	6	6	4	7	6	11	10	7	15	9	4	7	6	4	2	5	11	15	3	100.00	9.40	3.79
ESE058_1	21	5	3	1	5	1	4	8	6	4	5	5	7	6	2	5	6	3	6	1	11	100.00	3.88
ESE001_1	22	3	4	3	4	2	6	6	5	8	4	6	5	5	7	3	3	4	4	2	5	4	100.00
GALQ01085289_1	23	8	6	3	5	2	4	3	4	7	3	1	2	6	2	2	5	2	7	0	9	4	5
PRR1	24	2	2	2	4	2	1	2	1	2	2	2	2	3	2	1	3	2	1	5	2	4	4
ESO003_1	25	8	1	9	6	2	12	6	7	10	8	2	4	6	3	4	3	5	6	4	8	6	7
phiCb5	26	2	0	4	3	4	2	1	3	2	3	2	1	2	1	2	2	1	1	1	1	2	2
AP205	27	3	2	2	1	2	1	2	1	2	1	2	1	3	5	2	5	3	4	3	3	2	3
Wenling2_1	28	4	1	2	6	3	5	1	4	2	5	3	4	4	2	3	1	4	2	4	7	5	3
Hgal1	29	1	2	1	2	1	3	1	2	2	1	1	2	3	2	2	2	1	4	6	3	5	2
phageM	30	4	1	3	1	3	2	1	5	2	4	1	2	1	2	3	3	2	3	1	1	2	3
Wenzhou6_2	31	4	3	5	4	5	5	3	3	5	4	6	2	5	4	3	4	4	2	2	3	5	5
Hubei12_3	32	5	4	3	6	5	4	4	2	4	3	5	2	1	4	3	5	2	2	2	5	4	4
ESE011	33	2	1	2	2	3	1	2	3	3	4	3	4	2	3	5	3	3	2	2	5	4	4
PP7	34	5	2	1	1	2	6	4	3	2	4	2	5	5	4	1	5	3	1	2	5	5	3
AVE017	35	2	1	3	4	7	5	7	3	9	4	3	3	3	4	2	6	6	4	2	7	5	4
MS2	36	0	1	4	4	4	8	4	2	10	2	4	4	5	4	1	2	7	5	4	8	4	3
ESE000_1	37	5	1	1	3	3	7	6	4	8	4	3	4	8	4	4	7	5	5	2	6	6	5
Beihai22_3	38	3	1	4	4	3	4	5	1	3	2	3	4	2	3	3	4	3	3	1	5	4	3
AIN002	39	10	3	1	5	3	7	8	4	7	6	5	6	4	4	3	4	5	5	2	4	11	4
EMM000_2	40	1	2	3	3	3	5	1	4	4	2	4	3	1	2	1	3	6	2	3	3	4	2
Hubei7_1	41	6	1	4	8	3	5	2	5	6	4	2	8	5	6	3	4	9	8	2	10	8	7
GALQ010911402_4	42	3	2	7	3	4	4	3	4	4	5	3	3	3	3	1	2	4	6	3	9	1	6
Beihai9_2	43	2	3	3	2	4	3	4	3	2	3	3	3	3	2	1	3	3	3	6	3	3	4

	#	GALQ01085289_1	PRR1	ESO003_1	phiCb5	AP205	Wenling2_1	Hgal1	phageM	Wenzhou6_2	Hubei12_3	ESE011	PP7	AVE017	MS2	ESE000_1	Beihai22_3	AIN002	EMM000_2	Hubei7_1	GALQ010911402_4	Beihai9_2
		23	24	25	26	27	28	29	30	31	32	33	34	35	36	37	38	39	40	41	42	43
Beihai5	1	7.84	1.45	5.59	0.90	2.52	3.96	0.65	3.28	3.74	5.00	2.00	3.97	1.42	0.00	5.00	2.70	10.00	0.96	5.22	2.10	1.32
ESE027_1	2	9.38	2.17	0.70	0.00	2.70	1.56	1.87	1.22	3.95	6.15	1.64	2.17	0.99	0.96	1.11	1.37	3.03	3.08	0.96	1.90	2.80
Beihai15_1	3	4.69	2.38	7.03	2.40	3.08	4.44	1.05	4.35	9.80	6.12	3.92	1.18	4.76	5.33	1.03	8.33	0.88	7.14	3.81	9.46	3.19
EMS003_2	4	6.76	4.44	4.69	1.69	1.41	10.91	1.90	1.27	5.88	10.71	3.57	1.28	4.30	3.81	3.90	6.35	5.32	5.36	9.30	2.97	1.92
Beihai35_2	5	3.51	2.63	1.56	2.50	3.51	8.33	1.14	4.84	11.63	12.82	6.98	2.56	10.61	5.13	3.41	7.14	2.88	8.57	3.12	5.33	4.60
Beihai2	6	3.05	0.68	9.38	0.85	0.78	4.59	1.85	1.47	4.63	3.64	0.88	4.41	4.31	6.25	5.83	3.67	5.47	4.59	4.13	3.25	1.86
ESE018_1	7	2.78	1.63	4.69	0.47	1.90	1.16	0.72	0.88	3.41	4.65	2.22	3.39	6.73	3.45	5.71	5.88	6.90	1.16	1.98	2.70	2.90
Beihai13_3	8	5.80	0.94	4.90	1.63	1.16	5.97	1.71	6.02	3.70	2.82	4.84	3.00	2.83	1.79	4.60	1.22	4.21	5.88	4.95	3.45	2.63
Wenzhou1_2	9	6.42	1.39	7.19	0.88	1.61	1.85	1.27	1.59	4.50	3.70	2.88	1.45	7.50	8.33	6.61	2.86	5.47	3.81	5.04	3.28	1.27
Sanxia1_ORF4	10	3.95	1.79	5.59	1.51	1.08	6.41	0.79	4.12	4.76	3.90	5.06	3.54	3.64	1.82	3.64	2.22	5.61	2.47	3.51	4.55	2.38
EMS016_1	11	1.79	2.33	1.46	1.20	2.99	6.67	1.02	1.52	11.76	10.87	6.25	2.27	3.49	4.08	3.33	4.84	5.32	8.33	1.98	3.16	3.12
Beihai8	12	2.47	1.74	2.80	0.51	1.04	5.13	1.54	2.06	2.38	2.60	5.33	4.85	2.54	3.28	4.08	4.55	6.12	3.70	7.48	2.46	2.33
Beihai7	13	7.41	2.65	4.20	1.00	3.19	5.13	2.34	1.01	5.95	1.30	2.60	4.95	2.54	4.10	8.16	2.27	4.08	1.23	4.67	2.46	2.36
Beihai9_1	14	2.63	2.17	2.34	0.56	6.76	3.64	1.89	2.47	6.67	7.41	4.84	4.26	4.26	3.77	4.65	4.41	4.26	3.45	6.38	2.97	1.89
AIN003_2	15	3.77	1.32	3.03	1.30	3.51	7.69	2.30	5.45	6.12	7.32	15.15	1.30	2.60	1.15	4.76	5.66	3.26	2.50	3.45	1.14	1.18
Beihai32_4	16	10.20	4.23	2.24	1.27	9.80	2.56	2.35	5.45	7.69	12.50	7.89	7.14	7.89	2.27	8.97	8.51	4.40	7.69	4.40	2.35	3.57
ESE029_1	17	1.83	1.57	3.91	0.47	2.80	4.44	0.71	1.75	4.30	2.22	3.26	2.48	6.38	6.60	4.27	3.37	3.88	6.82	8.04	3.88	2.14
Beihai34_1	18	6.73	0.70	4.20	0.44	3.28	1.83	2.60	2.40	1.67	1.82	1.92	0.71	3.45	4.31	4.00	2.48	3.88	1.83	6.61	5.04	1.96
phageC1	19	0.00	6.85	2.17	0.50	4.05	4.94	8.57	0.98	2.13	2.35	2.41	2.30	1.65	3.01	1.49	1.03	1.36	3.57	1.44	2.31	8.70
Changjiang3_1	20	8.41	1.42	5.76	0.44	2.46	6.80	1.92	0.79	2.75	4.81	4.81	3.68	6.80	7.77	4.44	4.85	2.80	2.91	7.69	8.74	1.94
ESE058_1	21	5.63	4.00	4.23	1.07	2.50	7.25	4.39	2.35	6.10	5.71	6.25	5.75	4.72	3.54	6.98	5.26	11.96	5.80	8.08	0.87	2.65
ESE001_1	22	5.38	3.57	5.47	1.02	3.23	4.23	1.61	3.06	7.04	5.56	5.33	2.63	3.85	2.59	5.32	3.75	3.92	2.82	6.80	5.31	3.25
GALQ01085289_1	23	100.00	3.49	2.80	0.60	4.55	4.17	3.09	0.00	4.92	9.80	4.17	2.30	3.49	3.49	5.38	6.45	5.94	6.25	3.77	2.22	2.11
PRR1	24	3	100.00	1.20	0.53	9.09	2.94	7.25	1.11	5.00	5.80	1.41	7.35	1.90	1.71	2.61	3.85	3.10	1.45	3.31	0.88	15.94
ESO003_1	25	4	2	100.00	3.17	3.40	6.25	2.21	3.25	3.12	3.12	1.53	1.95	6.25	4.48	6.77	3.12	2.84	3.91	6.25	6.98	1.67
phiCb5	26	1	1	8	100.00	1.18	2.67	0.50	4.32	3.05	1.30	1.38	0.53	2.12	1.49	1.50	1.21	0.93	0.66	0.48	0.50	1.02
AP205	27	3	5	5	2	100.00	4.08	7.25	2.86	3.23	8.00	1.92	7.14	3.49	3.06	4.17	3.39	2.73	4.08	1.94	1.05	8.82
Wenling2_1	28	2	2	8	4	2	100.00	3.75	11.54	12.82	16.67	12.12	1.43	7.46	3.80	6.25	4.65	5.26	10.34	5.75	6.58	2.60
Hgal1	29	3	5	4	1	5	3	100.00	0.99	3.26	2.44	1.20	3.66	2.59	1.56	1.54	1.09	1.39	1.25	2.21	0.00	13.64
phageM	30	0	1	5	6	2	6	1	100.00	4.55	5.36	6.82	1.10	4.40	0.97	4.04	1.49	2.63	5.66	2.70	2.94	2.04
Wenzhou6_2	31	3	4	4	5	2	5	3	3	100.00	19.51	4.26	6.10	10.96	7.06	6.45	6.12	3.96	10.26	4.21	4.88	5.49
Hubei12_3	32	5	4	4	2	4	5	2	3	8	100.00	13.51	4.23	5.71	7.32	6.17	6.67	4.26	9.09	3.49	1.28	4.94
ESE011	33	2	1	2	2	1	4	1	3	2	5	100.00	2.74	2.74	3.53	6.49	4.08	4.35	8.57	6.82	2.41	1.25
PP7	34	2	5	3	1	4	1	3	1	5	3	2	100.00	2.83	1.69	3.88	3.95	5.98	2.86	4.59	0.87	8.54
AVE017	35	3	2	8	4	3	5	3	4	8	4	2	3	100.00	32.00	5.08	5.80	2.22	4.69	3.57	9.46	2.59
MS2	36	3	2	6	3	3	3	2	1	6	6	3	2	24	100.00	2.31	6.17	0.70	6.58	1.63	7.59	1.56
ESE000_1	37	5	3	9	3	4	5	2	4	6	5	5	4	6	3	100.00	7.95	12.77	6.17	9.18	4.76	2.33
Beihai22_3	38	4	3	4	2	2	2	1	1	3	3	2	3	4	5	7	100.00	3.81	10.00	5.21	3.85	2.17
AIN002	39	6	4	4	2	3	5	2	3	4	4	4	7	3	1	12	4	100.00	6.12	10.28	2.11	2.10
EMM000_2	40	3	1	5	1	2	3	1	3	4	3	3	2	3	5	5	4	6	100.00	5.56	8.00	2.56
Hubei7_1	41	4	4	8	1	2	5	3	3	4	3	6	5	4	2	9	5	11	5	100.00	8.47	2.21
GALQ010911402_4	42	2	1	9	1	1	5	0	3	4	1	2	1	7	6	6	3	3	6	10	100.00	0.79
Beihai9_2	43	2	11	3	2	6	2	9	2	5	4	1	7	3	2	3	2	3	2	3	1	100.00

Upper comparison: Percent identity

min 1-30 max

Lower comparison: Identities

min 1-25 max

Supplementary Figure 4. Pairwise comparison of both classic and novel Sgls.
The primary structures of both classic and novel Sgls were compared using pairwise alignment. The percent identities are shown in the top half-diagonal and the number of identities per pair are shown in the bottom half-diagonal. Percent identities are scaled from red (1) to pink (>30) and amino acid identities are scaled from red(1) to pink (>25).

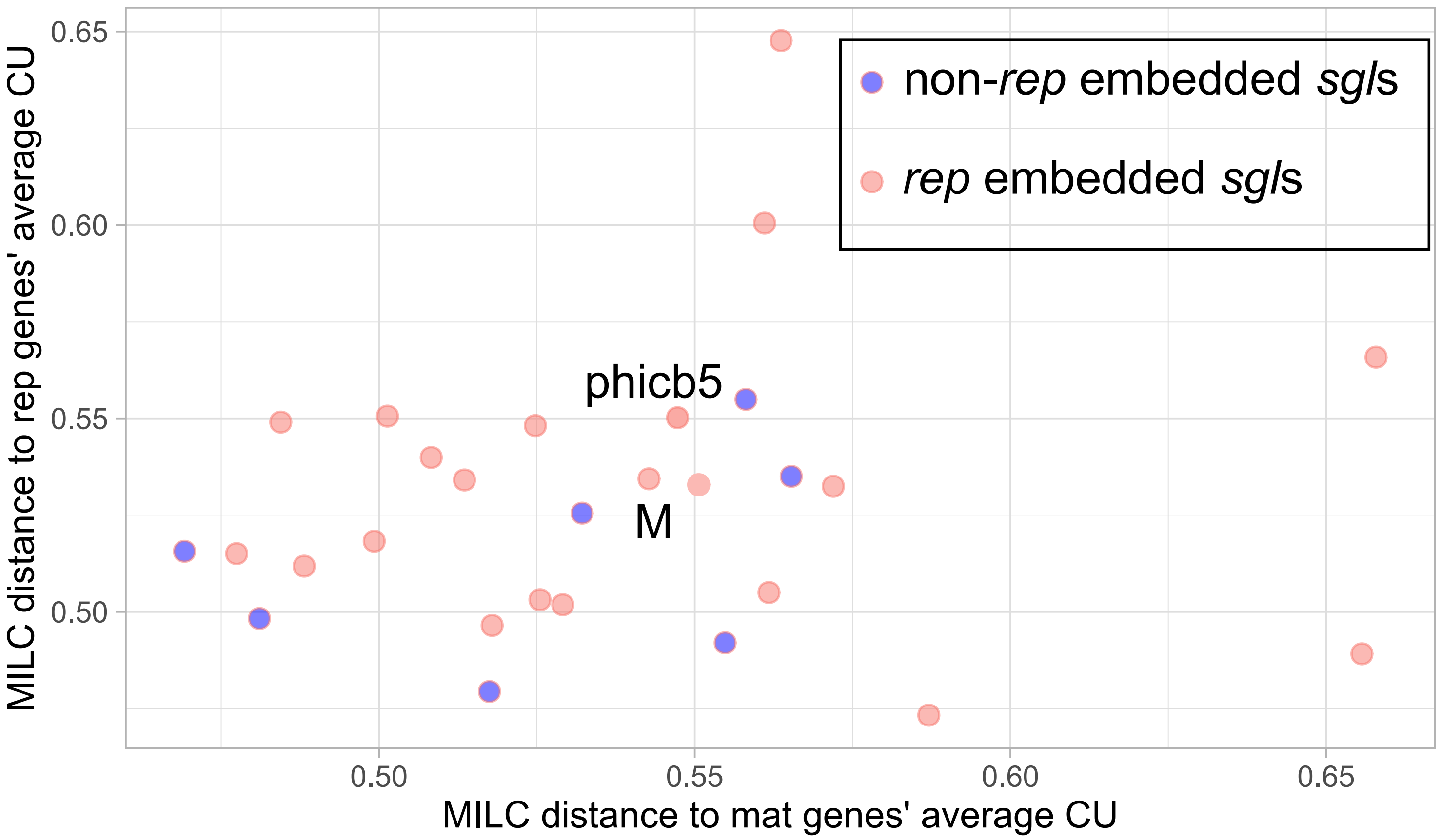

Supplementary Figure 5. Codon usage analysis of *mat* and *rep* genes.
Codon usage of *mat* and *rep* genes from genomes that have *rep*-embedded and non-*rep* embedded *sgl*s was calculated using Measure independent of length and composition (MILC). The MILC distance of a *rep* gene was plotted against the MILC distance of the corresponding *mat* gene.

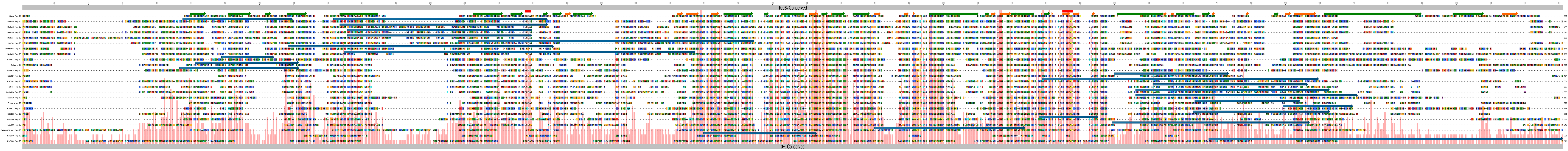

Supplementary Figure 6. Alignment of Replicases from genomes with functional *rep* embedded *sgls*.
The primary structures of Replicases with at least one embedded functional *sgl* were aligned along with Qβ Rep (at the top of the alignment). The corresponding *sgl*s are mapped onto the primary structures as blue arrows. The secondary structure elements of Qβ Rep are shown above the alignment as green and orange arrows. The sequence conservation is overlaid on the alignment as pink lines (0% conservation at bottom and 100% at the top) and conserved motifs"GPGA" and "FRESCG" are marked as red boxes above the alignment.

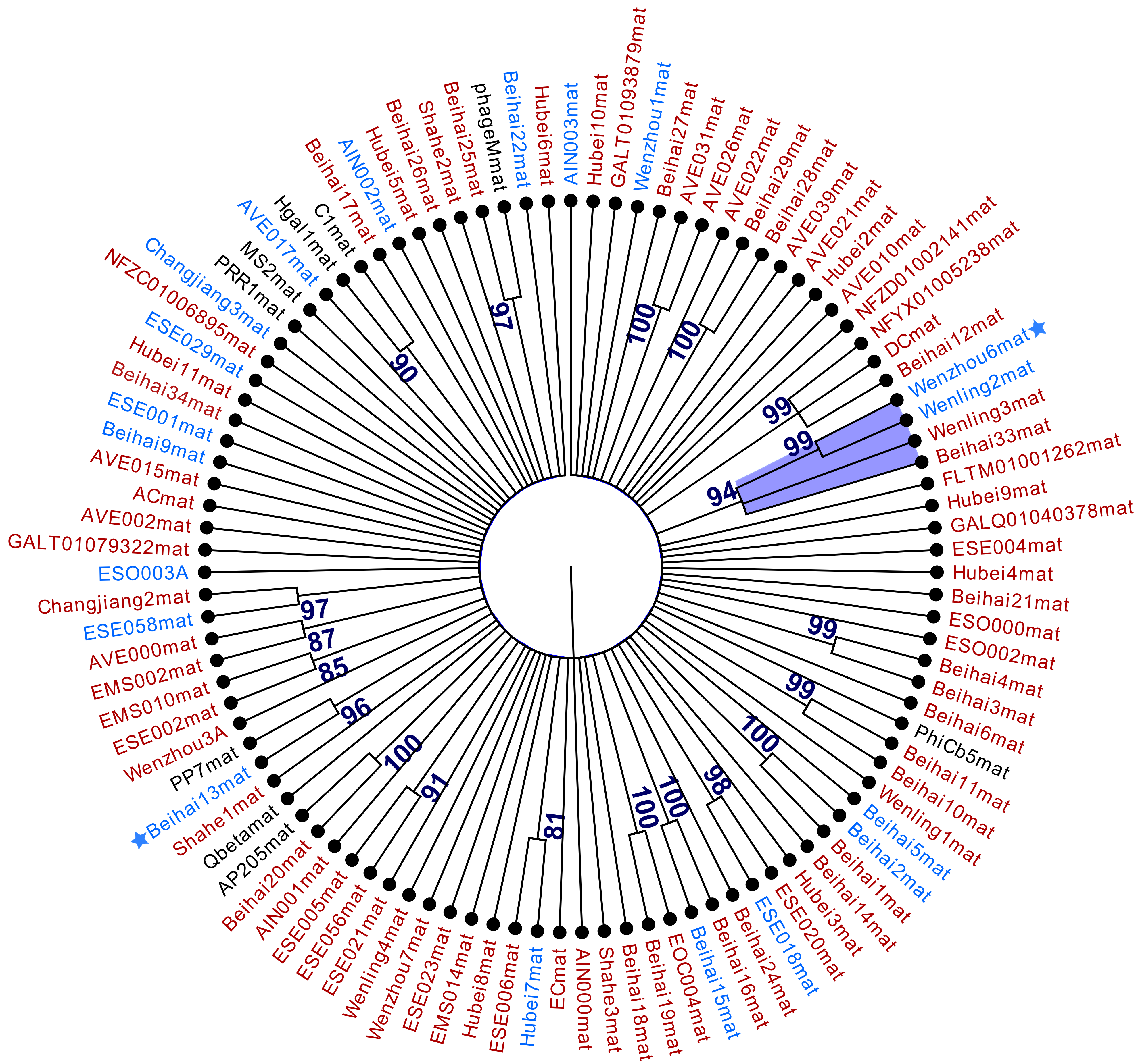

Supplementary Figure 7. Circular cladogram based on full length Mat sequences.
Cladogram of ssRNA phages based on the Mat sequences. Nodes with bootstrap values >80 are shown (resampling n = 1000). The genomes with functional sgls are highlighted in blue and the ones with mat-embedded sgls marked with an Asterix. Nodes with two or more levels are grouped as clades and highlighted.

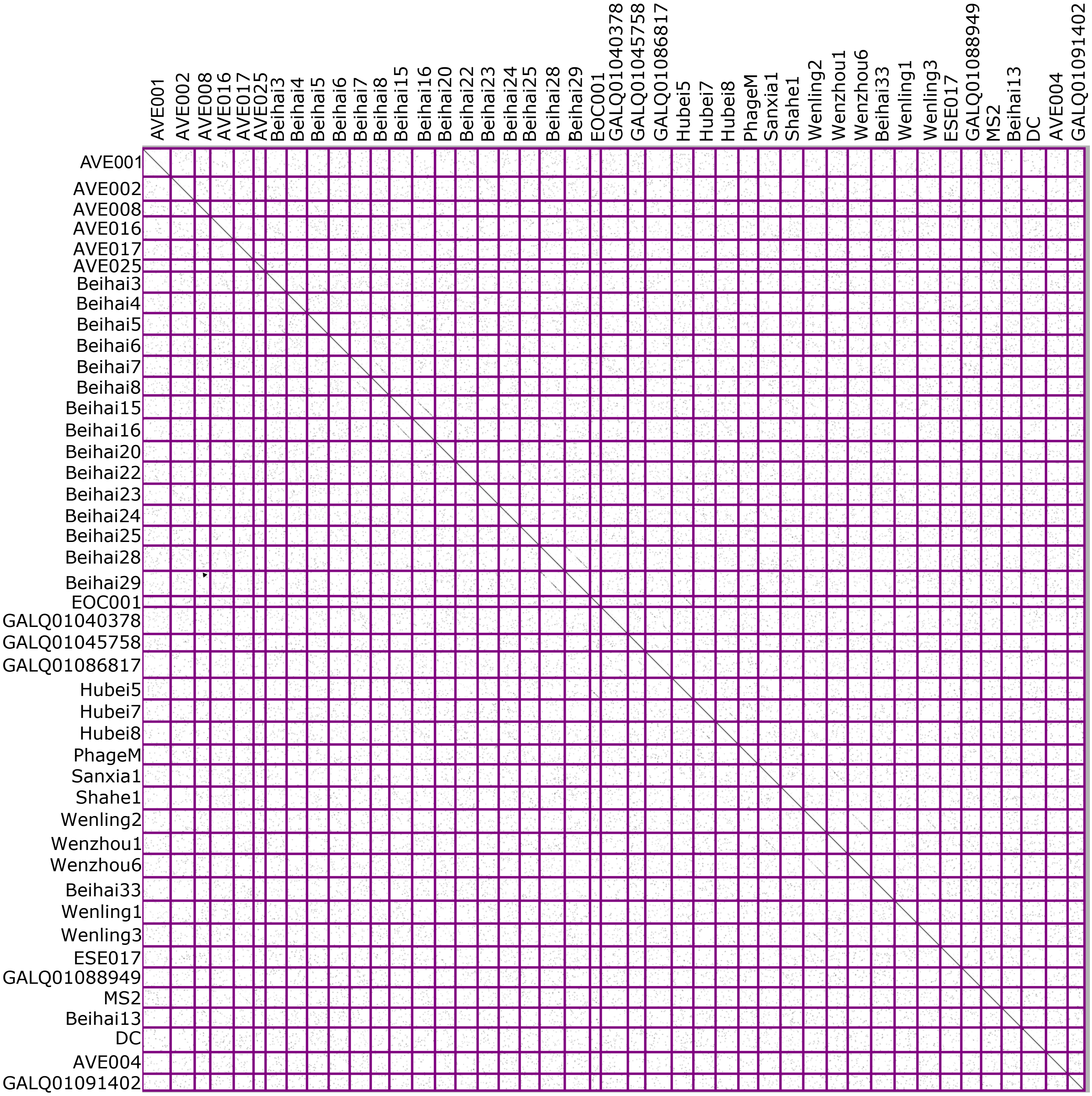

Supplementary Figure 8. MIST plot of genomes.

Nucleotide sequence dot plot of genomes (n = 44 genomes) that are classified as related based on the Replicase phylogeny (Figure. 3B).

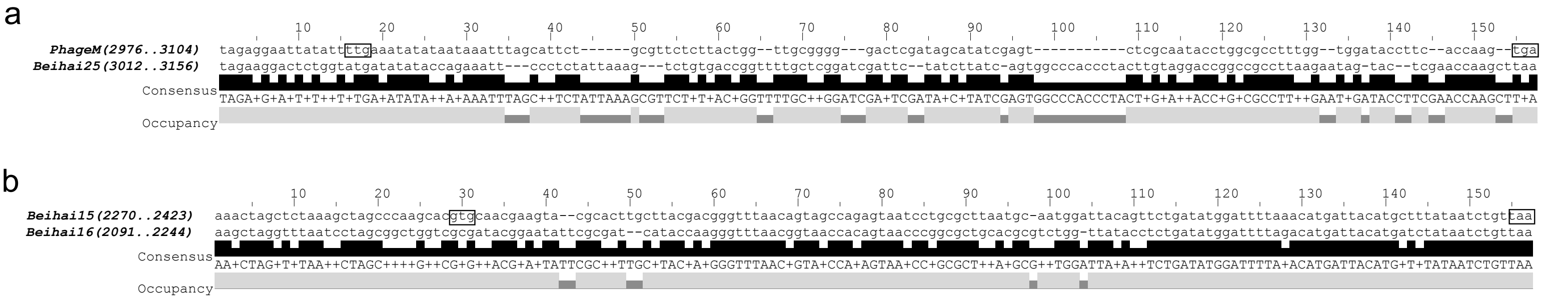

Supplementary Figure 9. Sequence comparison of regions that gave rise to *sgl* genes.
(**a**) Nucleotide alignment showing the *sgl* sequence divergence between phage M and Beihai25. The start and stop codons of the functional *sgl*s are boxed. (**b**) Nucleotide alignment between Behia15 and Beihai16.

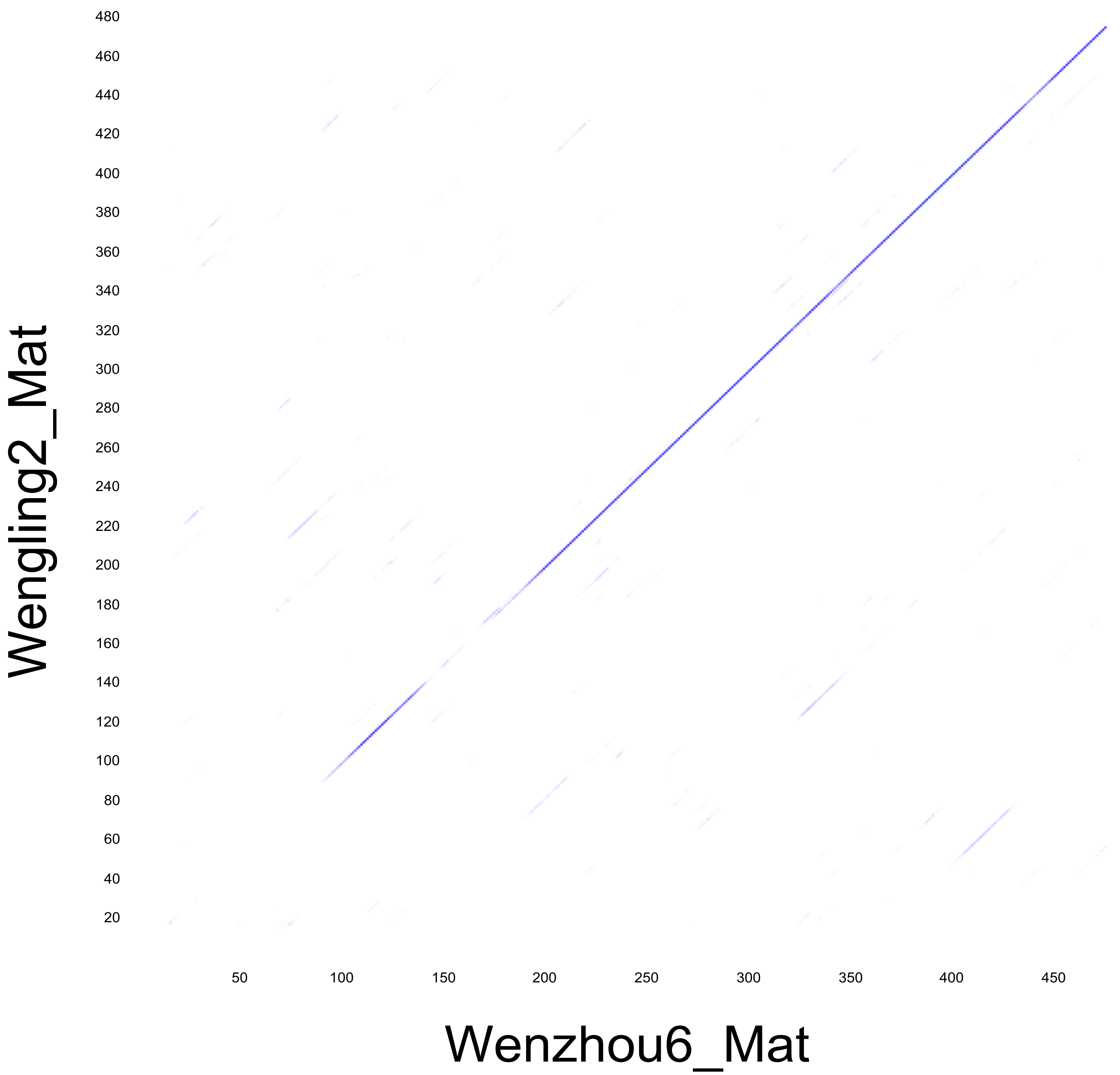

Supplementary Figure 10. Dot plot between the Mat proteins from Wenzhou6 and Wenling2.
The full length Mat protein sequences from Wenzhou6 and Wenling2 (n =2) were plotted against each other to generate a dot plot. The regions that match are shown as diagonal blue lines.

Supplementary References

1 Guzman, L. M., Belin, D., Carson, M. J. & Beckwith, J. Tight regulation, modulation, and high-level expression by vectors containing the arabinose P_{BAD} promoter. *J. Bacteriol* **177**, 4121-4130, doi:10.1128/jb.177.14.4121-4130.1995 (1995).

2 Chamakura, K. R. *et al.* A viral protein antibiotic inhibits lipid II flippase activity. *Nat Microbiol* **2**, 1480-1484, doi:10.1038/s41564-017-0023-4 (2017).

3 Chamakura, K. & Young, R. Phage single-gene lysis: Finding the weak spot in the bacterial cell wall. *J Biol Chem* **294**, 3350-3358, doi:10.1074/jbc.TM118.001773 (2019).

4 Krishnamurthy, S. R., Janowski, A. B., Zhao, G., Barouch, D. & Wang, D. Hyperexpansion of RNA Bacteriophage Diversity. *PLoS Biol* **14**, e1002409, doi:10.1371/journal.pbio.1002409 (2016).
