# Supplementary material for: Rapid de novo evolution of lysis genes in single-stranded RNA phages
Source: Nat Commun. 2020 Nov 26;11:6009. doi: 10.1038/s41467-020-19860-0 (PMC7693330; doi:10.1038/s41467-020-19860-0)
Supplement: Supplementary file 3 — Description of Additional Supplementary Files [file 41467_2020_19860_MOESM3_ESM.pdf]

Description of Additional Supplementary Files

File Name: Supplementary Data 1
Description: Information about the phage genomes and SgI clones used in this study.

File Name: Supplementary Data 2
Description: Synthetic DNA blocks and primers used in this study.
