# Supplementary material for: Rapid de novo evolution of lysis genes in single-stranded RNA phages
Source: Nat Commun. 2020 Nov 26;11:6009. doi: 10.1038/s41467-020-19860-0 (PMC7693330; doi:10.1038/s41467-020-19860-0)
Supplement: Supplementary file 7 — Reporting Summary [file 41467_2020_19860_MOESM7_ESM.pdf]

nature research

Corresponding author(s): Ry Young

Last updated by author(s): Oct 29, 2020

Reporting Summary

Nature Research wishes to improve the reproducibility of the work that we publish. This form provides structure for consistency and transparency in reporting. For further information on Nature Research policies, see our Editorial Policies and the Editorial Policy Checklist.

Statistics

For all statistical analyses, confirm that the following items are present in the figure legend, table legend, main text, or Methods section.

n/a	Confirmed	
☐	☒	The exact sample size (*n*) for each experimental group/condition, given as a discrete number and unit of measurement
☒	☐	A statement on whether measurements were taken from distinct samples or whether the same sample was measured repeatedly
☒	☐	The statistical test(s) used AND whether they are one- or two-sided<br>*Only common tests should be described solely by name; describe more complex techniques in the Methods section.*
☒	☐	A description of all covariates tested
☒	☐	A description of any assumptions or corrections, such as tests of normality and adjustment for multiple comparisons
☒	☐	A full description of the statistical parameters including central tendency (e.g. means) or other basic estimates (e.g. regression coefficient) AND variation (e.g. standard deviation) or associated estimates of uncertainty (e.g. confidence intervals)
☒	☐	For null hypothesis testing, the test statistic (e.g. *F*, *t*, *r*) with confidence intervals, effect sizes, degrees of freedom and *P* value noted<br>*Give P values as exact values whenever suitable.*
☒	☐	For Bayesian analysis, information on the choice of priors and Markov chain Monte Carlo settings
☒	☐	For hierarchical and complex designs, identification of the appropriate level for tests and full reporting of outcomes
☒	☐	Estimates of effect sizes (e.g. Cohen's *d*, Pearson's *r*), indicating how they were calculated

Our web collection on statistics for biologists contains articles on many of the points above.

Software and code

Policy information about availability of computer code

Data collection	No software was used for data collection.
Data analysis	CLC Genomics Workbench Version 8.0.1 (Qiagen) was used to generate sequence alignments, cladograms, protein dot plots, pairwise alignments of Sgls, and to perform local BLAST of Sgls. SnapGene version 5.1 (GSL Biotech LLC) was used to annotate the sgl gene candidates in ssRNA phage genomes. Transmembrane domains (TMDs) were predicted using TMHMM server, v.2.0 http://www.cbs.dtu.dk/services/TMHMM/. Kaleidagraph 4.03 (Synergy Software) was used to plot the lysis profiles. UCSF Chimera package was used to render the structure of Qbeta replicase beta subunit. MIST plot and nucleotide dot plots were generated using Galaxy Tools available at https://doi.org/10.5281/zenodo.4048782. RStudio Version 1.3.1073 used for Codon Usage analysis and for generating Figure 5a.

For manuscripts utilizing custom algorithms or software that are central to the research but not yet described in published literature, software must be made available to editors and reviewers. We strongly encourage code deposition in a community repository (e.g. GitHub). See the Nature Research guidelines for submitting code & software for further information.

Data

Policy information about availability of data

All manuscripts must include a data availability statement. This statement should provide the following information, where applicable:

- Accession codes, unique identifiers, or web links for publicly available datasets
- A list of figures that have associated raw data
- A description of any restrictions on data availability

All data generated and analyzed during the current study are available from the corresponding author upon reasonable request. The source data for Figures 3b, 3c, and 3e, Figures 4d and 4e, and Supplementary Figure3a-d are available in the Source data file. The ssRNA phage genome sequences used in this study were sourced from previously deposited and/or published sources18,19. Of the 244 genomes, 96 have GenBank accession numbers (see Supplementary Data 1) and the rest of

the genomes are available at https://doi.org/10.1371/journal.pbio.1002409.s001 . The structure of Qβ replicase beta subunit was obtained from Protein Data Bank (PDB:4R71).

Field-specific reporting

Please select the one below that is the best fit for your research. If you are not sure, read the appropriate sections before making your selection.

☒ Life sciences ☐ Behavioural & social sciences ☐ Ecological, evolutionary & environmental sciences

For a reference copy of the document with all sections, see nature.com/documents/nr-reporting-summary-flat.pdf

Life sciences study design

All studies must disclose on these points even when the disclosure is negative.

Sample size	The number of ssRNA phage genomes used in this study were the total available ssRNA phage genomes in the NCBI database as of September 2019.
Data exclusions	No data were excluded.
Replication	The experiments were repeated at least three times and all attempts at replication were successful.
Randomization	Randomization is not relevant to our study as we included all 244 genomes in our analysis.
Blinding	Blinding is not relevant to the current study. Candidate lysis genes were scored on binary phenotypes i.e., growth or no-growth of cells.

Reporting for specific materials, systems and methods

We require information from authors about some types of materials, experimental systems and methods used in many studies. Here, indicate whether each material, system or method listed is relevant to your study. If you are not sure if a list item applies to your research, read the appropriate section before selecting a response.

Materials & experimental systems

n/a	Involved in the study
☒	☐ Antibodies
☒	☐ Eukaryotic cell lines
☒	☐ Palaeontology and archaeology
☒	☐ Animals and other organisms
☒	☐ Human research participants
☒	☐ Clinical data
☒	☐ Dual use research of concern

Methods

n/a	Involved in the study
☒	☐ ChIP-seq
☒	☐ Flow cytometry
☒	☐ MRI-based neuroimaging
