# Supplementary material for: Rapid de novo evolution of lysis genes in single-stranded RNA phages
Source: Nat Commun. 2020 Nov 26;11:6009. doi: 10.1038/s41467-020-19860-0 (PMC7693330; doi:10.1038/s41467-020-19860-0)
Supplement: Supplementary file 2 — Peer Review File [file 41467_2020_19860_MOESM2_ESM.pdf]

Editorial Note: This manuscript has been previously reviewed at another journal that is not operating a transparent peer review scheme. The manuscript was considered suitable for publication without further review at *Nature Communications.*
